# Genetic Variants, Bioactive Compounds, and PCSK9 Inhibitors in Hyper-LDL-Cholesterolemia: A GWAS and In Silico Study on Cardiovascular Disease Risk

**DOI:** 10.3390/nu17091411

**Published:** 2025-04-23

**Authors:** Meiling Liu, Junyu Zhou, Sunmin Park

**Affiliations:** 1 Department of Chemical Engineering, Shanxi Institute of Science and Technology, Jincheng 048000, China; liumeiling@sxist.edu.cn; 2 Department of Food and Nutrition, Obesity/Diabetes Research Center, Hoseo University, Asan 31499, Republic of Korea; 3 Institute of Advanced Clinical Medicine, Peking University, Beijing 100191, China; zjy888zjy888@gmail.com; 4 Department of Bioconvergence, Hoseo University, Asan 31499, Republic of Korea

**Keywords:** cardiovascular diseases, serum LDL-C concentration, polygenic risk scores, exercise

## Abstract

**Background:** Hyper-LDL-cholesterolemia is a key contributor to cardiovascular diseases (CVDs), and both genetic predisposition and lifestyle influence it. This study aimed to develop personalized strategies for managing hyper-LDL-cholesterolemia by integrating polygenic risk scores (PRSs), genetic variants, and bioactive compound interactions, leveraging a precision medicine approach. **Methods:** A cohort of 58,701 Korean adults, including 8966 individuals with hyper-LDL-cholesterolemia (LDL ≥ 160 mg/dL) or undergoing treatment with hypocholesterolemic agents, was analyzed to investigate the interplay between genetic risk and lifestyle factors. The PRS was constructed using three key variants: PCSK9 (rs151193009), CELSR2 (rs11102967), and APOE (rs7412). Gene–lifestyle interactions were assessed, focusing on energy intake and physical activity. Computational molecular docking was utilized to investigate how bioactive compounds differentially interact with the wild-type and mutant forms of PCSK9 (Arg93Cys) and APOE (Arg176Cys), focusing on variations in binding affinity. **Results:** Hyper-LDL-cholesterolemia was associated with a 1.3-fold increased risk of CVD. The PRS showed a significant association with a 3.45-fold higher likelihood of developing both elevated LDL cholesterol and reduced HDL cholesterol levels. Lifestyle interactions revealed that high energy intake and physical inactivity significantly amplified the genetic risk (*p* < 0.05). In silico analysis demonstrated that bioactive compounds, notably prodelphinidin trimer, exhibited enhanced binding affinity with wild-type PCSK9 (Arg93Cys), while several compounds preferentially targeted the mutated PCSK9, suggesting potential avenues for genotype-specific therapies. **Conclusions:** This study emphasizes the combined influence of genetic predispositions and lifestyle behaviors on developing hyper-LDL-cholesterolemia, and highlights potential bioactive compounds as personalized therapeutic targets. By integrating genomic data, lifestyle analysis, and molecular docking, this research provides a foundation for precision interventions tailored to an individual’s genetic and metabolic profile, paving the way for more effective and personalized management of dyslipidemia and associated CVD risk.

## 1. Introduction

Hypercholesterolemia, particularly elevated levels of low-density lipoprotein cholesterol (LDL-C), remains a significant risk factor for cardiovascular disease (CVD). Although statins have been the first choice for lowering serum LDL-C concentrations in the past few decades [1], showing efficacy and laying a solid foundation for treatment, people are beginning to pay more attention to the potential side effects and individual differences in their long-term use. This concern has prompted the scientific community to continuously explore new alternative treatments that may be more suitable for specific patient groups, aiming to achieve safer and more effective LDL-C management [2]. One promising approach focuses on inhibiting the enzyme proprotein convertase subtilisin/kexin type 9 (*PCSK9*), a protease crucial in cholesterol metabolism [3]. *PCSK9* regulates serum LDL-C concentrations by binding to LDL-receptors (LDL-Rs). This prevents LDL-C from binding to its receptors, and promotes LDL-R degradation, thereby leading to increased serum LDL-C concentrations [4,5]. Inhibition of *PCSK9* results in higher availability of LDL-Rs, enhancing LDL-C clearance from the bloodstream.

Genetic studies underscore the importance of *PCSK9* in lipid metabolism. Various mutations in the *PCSK9* gene that affect the secretion and function of the enzyme have been identified. PCSK9 loss-of-function mutations, including G236S, S462P, and C679X, are known to impair its secretion [6], whereas gain-of-function variants like E32K lead to increased secretion [7]. Of particular interest is the R93C mutation, a loss-of-function variant that is negatively correlated with serum total cholesterol and LDL-C concentrations [8]. These genetic results highlight *PCSK9* as a potential therapeutic target for managing hyper-LDL-cholesterolemia. Recent clinical studies have demonstrated that combining statins with *PCSK9* inhibitors can significantly improve cardiovascular outcomes [9]. However, currently available *PCSK9* inhibitors are injectables and have limitations, such as side effects and high costs, in addition to the discomfort associated with parenteral administration [9]. These challenges highlight the need for continued research into effective cholesterol-lowering drugs with minimal side effects and, ideally, improved oral bioavailability.

Importantly, the polygenic risk score (PRS) generated from interacted genetic variants may synergistically modulate *PCSK9* function and serum LDL-C concentration. The complex interplay between genetics and lifestyle factors in hyper-LDL-cholesterolemia suggests that the PRS, including that of *PCSK9* and its functionally linked genes, not only modulates serum LDL-C concentrations directly, but may also interact with certain lifestyle factors to significantly and synergistically contribute to elevated serum LDL-C concentrations. Furthermore, these interactions between genetics and lifestyle factors could potentially affect the efficacy of *PCSK9*, highlighting the need for personalized treatment approaches.

Given that previous studies have revealed the side effects and individual differences associated with long-term use of statins, as well as the limitations of existing PCSK9 inhibitors, such as discomfort, significant side effects, and the high cost of parenteral administration, and considering the complex interaction between genetic factors and lifestyle in regulating LDL-C concentration, this study takes a unique approach. The primary objective of the present study was to establish personalized strategies for managing serum LDL cholesterol levels by integrating genetic, lifestyle, and molecular data. To achieve this, we pursued three specific aims: (1) to identify polygenic variants associated with hyper-LDL-cholesterolemia using genome-wide association study (GWAS) and generalized multifactor dimensionality reduction (GMDR) analyses; (2) to investigate interaction between the PRS derived from these variants and lifestyle factors in modulating serum LDL-C levels; and (3) to utilize in silico modeling to explore potential bioactive compounds targeting *PCSK9* missense mutations. Through this integrative approach, we aimed to elucidate gene–lifestyle interactions and develop tailored nutritional strategies for hyper-LDL-cholesterolemia management. Each sub-objective was systematically addressed through rigorous statistical analyses and interaction studies, as well as computational modeling. This multifaceted strategy represents a novel approach to personalized nutrition for cardiovascular health, with the ultimate goal of reducing CVD risk through precision medicine strategies.

## 2. Methods

### 2.1. Participants

Between 2004 and 2013, 58,701 participants (20,293 male and 38,408 female) voluntarily participated in a hospital-based urban cohort as part of the Korea Genome and Epidemiology Study (KoGES). This study adhered to the ethical standards outlined in the Declaration of Helsinki. The research protocol received approval from the Institutional Review Boards of the Korea National Institutes of Health (KBP-2019-055) and Hoseo University (1041231-190902-BR-099-01). Written informed consent was obtained from all participants.

### 2.2. Participant Characteristics and Biochemical Measurements

Volunteers were interviewed regarding their health status, and data including their gender, age, education level, etc., were recorded. The mean age of men and women was 56.0 ± 0.06 and 52.5 ± 0.04 years, respectively. Education was classified as ≤middle school, high school, and ≥university. Household monthly income was categorized into three levels ($/month): low (<$2000), middle ($2000–$4000), and high (>$4000) [10]. Smoking status was classified as smoking (based on >100 cigarettes smoked in 6 months), former smoking (previously smoking but not for over 6 months), or non-smoking [11]. Regular physical activity was characterized as engaging in at least 150 min per week of moderate-intensity exercises, such as mowing the lawn, swimming, playing badminton, or playing tennis. Alcohol and coffee intake were also calculated using frequencies and amounts per time. Anthropometric and biochemical tests were carried out. Standardized procedures were used to measure body weight, height, and waist circumference [12]. Body mass index (BMI) was computed as the weight in kilograms divided by the height in meters squared (m^2^). Skeletal muscle and fat mass were estimated based on a previously developed XGBoost model [13]. The skeletal muscle index (SMI) was determined by dividing appendicular skeletal muscle mass (kg) by height in meters. Additionally, insulin resistance, assessed via the Homeostatic Model Assessment for Insulin Resistance (HOMA-IR), was predicted using a model from a prior study [14]. Blood was drawn from subjects fasting for more than 12 h. Plasma and serum were separated from the collected blood into the tubes with and without blood coagulators after centrifugation, and serum glucose, aspartate aminotransferase (AST), alanine aminotransferase (ALT), and lipid profiles were assessed in the samples by colorimetric kits (Asan Pharm., Seoul, Republic of Korea) [15]. Serum LDL concentration was calculated using the Friedewald equation [15]. Glycosylated hemoglobin (HbA1c) contents in the EDTA-treated blood were measured with an automated HbA1c analyzer (Arkray Inc., Kyoto, Japan). SBP and DBP were measured with a mercury sphygmomanometer in a sitting position and in a resting state.

### 2.3. Dietary Assessment and Analysis

Participants’ dietary patterns were assessed using a validated semi-quantitative food frequency questionnaire (SQFFQ) consisting of 106 items, aimed at capturing usual intake over the previous year. The SQFFQ’s validity had been previously confirmed by comparison with seasonal three-day food records [11]. Daily food consumption was calculated by combining frequency and portion size data. Nutritional analysis was performed using the CAN-Pro 2.0 software (Korean Nutrition Society, KNS) to estimate the daily intake of macronutrients, vitamins, and minerals.

For principal component analysis (PCA), food items were classified into 30 groups. Four distinct dietary patterns were derived using eigenvalues greater than 1.5 and varimax rotation, with factor loadings of 0.40 or higher deemed meaningful [11]. The identified patterns were labeled as balanced diet (BD), Western-style diet (WSD), plant-based diet (PBD), and high-white rice diet (HRD). These patterns were named according to their predominant food groups. The dietary inflammatory index (DII) was used to evaluate the inflammatory potential of each participant’s diet. It was calculated based on 38 nutritional components, with garlic, ginger, saffron, and turmeric excluded due to missing intake information. DII scores were derived by multiplying each component’s inflammatory weight by the corresponding daily intake, summing the results, and dividing the total by 100.

### 2.4. Hyper-LDL-Cholesterolemia Definition

Participants with lipid profiles were excluded. A serum LDL-C concentration of <160 mg/dL was considered normal, and ≥160 mg/dL was defined as hyper-LDL-cholesterolemia. Individuals receiving lipid-lowering medication (*n* = 2429) were included in the high-LDL-C group. Accordingly, the cohort was divided into control (*n* = 49,735; normal-LDL-C) and case (*n* = 8966; high-LDL-C) groups.

### 2.5. Genetic Variants Affecting Hyper-LDL-Cholesterolemia in Koreans

Single-nucleotide polymorphisms (SNPs) linked to serum LDL-C levels were identified, and gene–lifestyle interactions were assessed. Genetic variants associated with serum LDL-C in a Korean population were investigated through a genome-wide association study (GWAS) using PLINK software. Based on clinical data from the KoGES, participants were classified into high-risk and normal groups according to their serum LDL-C concentrations.

A GWAS comparing low-LDL and high-LDL groups in the urban hospital cohort was performed using PLINK version 2.0 (https://www.cog-genomics.org/plink/2.0/; accessed on 11 April 2024), after adjusting for confounders such as age, sex, education, income, daily energy intake, BMI, alcohol intake, physical activity, and smoking status. Genetic variants associated with hyper-LDL-cholesterolemia in Koreans were identified, and genes linked to these SNPs were explored through the SCAN annotation database (https://biit.cs.ut.ee/gprofiler/gost, accessed on 25 April 2024).

### 2.6. Genotyping and Quality Control

DNA was extracted from participants’ peripheral blood samples, and genotyping was performed using the Affymetrix Genome-Wide Human SNP Array 5.0 (Affymetrix, Santa Clara, CA, USA). The quality and accuracy of genotyping were assessed using the Bayesian Robust Linear Model with the Mahalanobis Distance Classifier (BRLMM) [15]. The exclusion criteria for DNA genotyping included the following: a high genotype deletion rate (≥4%), low genotyping accuracy (<98%), high heterozygosity (>30%), a minor allele frequency (MAF) <0.01, violations of Hardy–Weinberg equilibrium (*p* < 0.05), and gender bias [15].

### 2.7. PRS Affects Hyper-LDL-Cholesterolemia

Manhattan plots (Figure S1A) and quantile–quantile (Q-Q) plots (Figure S1B) were created using the “fastman” and “data.table” packages in R. GMDR was used to explore the interactions of PCSK9-specific SNPs with other genetic variants. Each risk allele of the selected genetic variants was counted as 1, and a non-risk allele was counted as 0. The sum of the risk alleles was used as the PRS, and 10 genetic variants were identified to build the best model of SNP–SNP interaction associated with the risk of high LDL-C, with *p* < 0.001 for the sign test of balanced accuracy (TEBA) and 10-fold cross-validation consistency (CVC), and adjusted for covariates such as age, gender, residence area, body mass index (BMI), education, and income (covariates 1), plus smoking, alcohol and energy intake, and physical activity (covariates 2) (Table S1). The non-risk, heterozygote, and risk alleles of SNPs associated with high LDL-C risk were assigned to 0, 1, and 2, respectively. The PRS was obtained by summing the number of risk alleles in the optimal model. The PRS of the best model was divided into 0–2, 3–4, and >4, which were called low-, medium-, and high-PRS groups. A high PRS indicates more risk alleles in the optimal genetic variation interaction model.

### 2.8. Interactions Between Genetic Variants and Lifestyle Factors Regarding Hyper-LDL-Cholesterolemia

The interaction between genetic variants and lifestyle factors in relation to serum LDL-C levels was assessed. The analysis included daily intake of energy, calcium, sodium, coffee, and alcohol, along with smoking status and physical activity levels. The dietary intake, smoking status, and physical activity were divided into high and low groups based on clinically meaningful values, such as the recommended intake among the dietary reference intakes (DRIs), and the 33rd or 66th percentiles of each parameter. The cutoff for high and low nutrient intake was based on the recommended intake by the General Dietary Guidelines for Koreans [16]. The cutoff values for each parameter are presented in the results section. Interactions were adjusted for variables such as survey year, residential area, age, gender, BMI, smoking status, alcohol consumption, coffee intake, daily physical activity, plasma total cholesterol, and serum glucose levels, unless the interaction parameter overlapped with any of the adjustment parameters.

### 2.9. Collection and Screening of Bioactive Compounds with Low Binding Energy from Foods with PCSK9

Among the three genes with genetic variants in the best model, the genes with missense genetic variants (*PCSK9*_rs151193009) were selected for analyzing the binding affinity to food components. The wild-type protein structure of a gene with a missense genetic variant was obtained from the UniProt program. The mutant gene was made by changing the amino acid corresponding to the genetic variant in the Swiss-PBD Viewer (SPDBV) program. Food compounds (*n* = 20,000) were downloaded from the fooDB website (https://foodb.ca/; accessed on 8 May 2024) and used as ligands. The proteins, hydrogenation, and Kolman charges of nonpolar hydrogen atoms were processed using MGLTools 1.5.6, a companion tool to the AutoDock Vina software. The original pdb file format was converted to pdbqt format, which is compatible with AutoDock Vina, to facilitate molecular docking of ligands. Active sites in the protein were identified using the Proteins Plus website (https://proteins.plus/, accessed on 15 May 2024), and the functional pocket containing the mutation site was chosen for docking. After docking each compound, those with low binding affinity were excluded. A lower binding energy indicates a stronger binding affinity to the active site [17].

### 2.10. Molecular Dynamics Simulation (MDS)

MDS analysis of the *PCSK9* complex was performed to validate the docking method used in this study. The screened molecules bound to the *PCSK9* complex were simulated using Discovery Studio software ver. 4.5 (Dassault System BIOVIA, San Diego, CA, USA). The CHARMM36 force field was applied to each molecular structure during preparation using “Simulation”, and the protein was solvated with the “Solvation” tool in Discovery Studio software ver. 4.5. Molecular dynamics simulation parameters for the protein within the solvent system were configured using the “Standard Dynamics Cascade”. Parameters such as acceleration time, equilibrium time, simulation sampling time, and simulation step size were set to 40 ps, 400 ps, 10,000 ps, and 2 fs, respectively, with all other parameters left at their default values [18]. Following a 10 ns simulation, the root–mean–square fluctuation (RMSF) and root–mean–square deviation (RMSD) of the PCSK9 protein complex were calculated to assess system flexibility and stability, respectively [19]. When the RMSD stabilized below 2.5 Å in the last 50 ns of simulation, the system was considered to have reached equilibrium. As for RMSF, calculations were performed for each residue of the protein. If the RMSF value of a residue was less than 1.5 Å, it indicated that the residue was structurally stable during the simulation process.

### 2.11. Statistical Analysis

Statistical analysis was conducted using the SAS software ver. 9.4 (SAS Inc., Cary, NC, USA). Participants with no serum LDL concentration measurement were excluded from the study, and there were no participants with no serum LDL concentration in the analysis. However, the missing data were minimal (<5% for any variable) in the variables we analyzed as independent variables, and they were excluded from each analysis in the SAS procedure. The Chi-square test was used to analyze the frequency distribution of the categorical variables. The means and standard deviations of continuous variables, such as age, BMI, waist circumference, and cholesterol level, were analyzed using two-sample *t*-tests. Significant differences between groups were determined by one-way analysis of variance (ANOVA) [20]. Tukey’s test was used for multiple comparisons among groups [20]. After adjusting for covariates, logistic regression analysis was conducted to investigate the association of PRS with the risk of hyper-LDL-cholesterolemia and the anthropometric and biochemical parameters related to serum LDL-C concentration. Logistic regression analysis was performed with the low-PRS group as the reference to estimate the adjusted odds ratio (OR) and 95% confidence interval (CI). Finally, the interaction between the SNP genotypes and lifestyle and their effects on serum LDL-C concentrations, after adjusting for covariates, were evaluated using multivariate interaction models. A *p* value of 0.05 was considered statistically significant.

## 3. Results

### 3.1. Metabolic Parameters According to Gender and Serum LDL-C Concentrations

**Figure 1:**
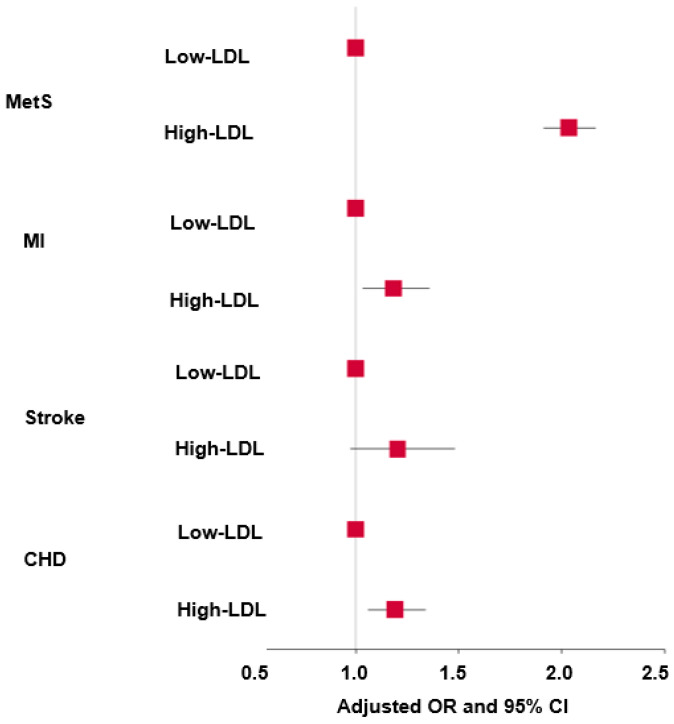
Adjusted odd ratios (ORs) and 95% confidence intervals (CIs) of hyper-LDL-cholesterolemia and metabolic syndrome and cardiovascular disease parameters. MI, myocardial infarction; CHD, cardiovascular disease, including MI and stroke. Covariates: BMI, residence area, gender, age, energy intake, alcohol intake, physical activity, smoking, and education.

After adjusting for covariates, there were significant differences in gender and age between the high-LDL and control (low-LDL) groups (Table 1). There was a difference in education level and income between the genders. Men had a higher level of education and income than women. However, there was no association between serum LDL-C concentrations and education levels or income (Table 1). BMI and waist circumference differed between the genders, and were found to be associated with the serum LDL-C concentrations. The high-LDL group exhibited higher BMI and waist circumference than the low-LDL group in both sexes (Table 1). Significant differences between genders were observed in SMI, fat mass, blood HbA1C levels, and serum concentrations of glucose, total cholesterol, HDL-C, LDL-C, and triglycerides (Table 1). In both sexes, all these parameters were elevated in the high-LDL-C group compared to the control group. The prevalence of insulin resistance was notably higher in men than in women (Table 1). As participants on lipid-lowering medication were included in the high-LDL group, all individuals in this group were classified as high-LDL. Among the men and women with high LDL, 40.5% and 30.7% had lipid-lowering medication, respectively (Table 1). For participants receiving lipid-lowering medication, the LDL levels were 97.4 ± 1.45 mg/dL for men and 105 ± 1.01 for women. The participants not receiving medication, the levels were 177 ± 0.70 mg/dL for men and 179 ± 0.41 mg/dL. SBP was notably higher in male participants and the high-LDL group. However, no significant differences were found for DBP (Table 1). The serum LDL-C concentrations of all the participants were positively correlated with MetS, myocardial infarction (MI), and coronary heart disease (CHD) risk, but not with stroke risk (Figure 1).

### 3.2. Nutrient Intake of Participants

Energy intake based on the estimated energy requirements was higher in women than in men (*p* < 0.001), and differences were also seen in the high-LDL group (*p* < 0.05 Table 2). Fiber intake was higher in women compared to men (Table 2). The intakes of calcium, sodium, vitamins C and D, and flavonoids were higher in women than in men, and the intakes of calcium, sodium, and vitamin D showed differences between the high-LDL-C and normal groups (Table 2). The mean DII value for both groups was negative, indicating that most participants consumed an anti-inflammatory diet. The DII of women was lower than that of men, indicating that the inflammatory components in the diets of the female group were lower than in that of men. The serum LDL-C concentrations differed among those consuming the WSD and RMD patterns across both genders (Table 2). The proportion of women who drank alcohol (*p* < 0.001) was significantly lower than that of men. Interestingly, the amount of alcohol consumption in the high LDL-C group was lower than that in the normal group for both genders (Table 2). The proportion of women who regularly participated in physical activity (*p* < 0.01) was significantly higher than that of men. Men smoked significantly more than women, and the proportion of smoking participants was slightly higher in the high-LDL-C group than in the normal group for both genders (Table 2). However, this was not statistically significant.

### 3.3. Distribution of Genetic Variants for Hyper-LDL-Cholesterolemia Risk in GWAS

Some genetic variants were significantly different between the high-LDL group and the low-LDL group, with a cutoff value of 160 mg/dL. Figure S1A shows a Manhattan plot of the *p*-values of the genetic variants associated with hyper-LDL-cholesterolemia. The Manhattan plot shows the statistical significance of each polymorphism in GWAS associated with increased LDL risk. The red line indicates *p* < 5 × 10^−8^, and Bonferroni correction is used. There are significant genetic variations in chromosomes 1, 2, 5, 8, 9, 16, 19, and 20. The Q-Q plot displays the quantile distribution of the logarithmic values of observed *p*-values on the *y*-axis and expected *p*-values on the *x*-axis. It illustrates the deviation in SNP associations from the GWAS with hyper-LDL-cholesterolemia. The genomic inflation factor was 1.016, close to 1 (Figure S1B). Both the Q-Q plot and the genomic inflation factor suggest minimal spurious associations between the SNP *p*-values and hyper-LDL-cholesterolemia, indicating that the SNPs were not significantly biased.

### 3.4. Genetic Variants Associated with Hyper-LDL-Cholesterolemia

Among the genetic variants of *PCSK9*, rs151193009 had the lowest *p*-value in the positive association with hyper-LDL-cholesterolemia. The genetic variants that interacted with the *PCSK9* genetic variant rs_151193009 were selected by GMDR. Cadherin EGF LAG Seven-Pass G-Type Receptor 2 (*CELSR2*)_rs11102967 and apolipoprotein E (*APOE*)_rs7412 interacted with rs151193009 to meet the criteria of trained balanced accuracy (TRBA) and cross-validation consistency (CVC) after adjusting for covariates. The three SNPs satisfied MAF (0.0151–0.0646) and HWE (*p* > 0.05) (Table 3). The ORs for hyper-LDL-cholesterolemia ranged from 0.324 to 0.689, with *p*-values ranging from 1.05 × 10^−68^ to 2.66 × 10^−14^, which met the Bonferroni correction (Table 3). The major alleles of these SNPs were risk alleles, which were known to have a high impact on the population (Table 3).

**Figure 2:**
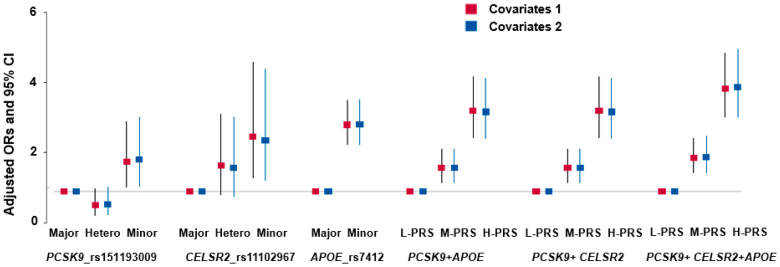
Adjusted odd ratios (ORs) and 95% confidence intervals (CIs) of hyper-LDL-cholesterolemia with genetic variants and their polygenic risk score (PRS). The PRS was calculated by summing the number of risk alleles of *PCSK9_rs151193009*, *CELSR2_rs11102967*, and *APOE_rs7412*. Covariates 1: age, gender, BMI, residence area, and education; covariates 2: the parameters in covariates 1 plus energy intake, alcohol intake, physical activity, and smoking status.

The PRS was determined by summing the risk alleles of the SNPs within the model. The low-, medium-, and high-PRS categories were defined based on the number of risk alleles. For the 3-SNP PRS, low, medium, and high PRSs were categorized as <3, 3–4, and >4, respectively. After adjusting for covariates, a high PRS for the three SNPs in the SNP–SNP interaction model showed a positive association with hyper-LDL-cholesterolemia (3.451; 2.771–4.298) (Table 4 and Figure 2). Among the ten PRS models evaluated, the three-SNP model—comprising *PCSK9* rs151193009, *CELSR2* rs11102967, and *APOE* rs7412—demonstrated the highest adjusted odds ratio (OR) for elevated LDL-C levels (Supplementary Table S1). Adding further SNPs did not improve model performance and, in some cases, attenuated the effect size. Therefore, the three-SNP PRS model was selected for subsequent analyses due to its optimal balance between statistical strength and biological relevance. The total cholesterol and serum HDL concentrations were positively correlated with the high-PRS, increasing by 2.452 and 1.439 times, respectively (Table 4), while serum triglyceride levels decreased. However, the PRS was not significantly associated with BMI, waist circumference, SMI, fat mass, blood glucose concentration, HbA1c, insulin resistance, and blood pressure (Table 4).

To assess the potential influence of lipid-lowering therapy on our results, we performed a sensitivity analysis excluding participants using lipid-lowering medications. The association trends between genetic variants, PRS categories, and LDL-C levels remained consistent, although the effect sizes were somewhat diminished. These findings suggest that our results are robust and not solely driven by individuals on pharmacological treatment (Supplementary Figure S2A).

As part of a sensitivity analysis, we repeated the primary analyses using a lower LDL-C threshold of ≥130 mg/dL (*n* = 20,857) to define hypercholesterolemia. The results remained consistent with those using the ≥160 mg/dL (*n* = 8966) threshold, and in several cases, the effect sizes were slightly larger (Supplementary Figure S2B). This suggests that the associations between polygenic variants, PRS levels, and LDL-C status are robust across clinically relevant definitions of hyper-LDL-cholesterolemia.

### 3.5. Interaction of PRS and Nutrient Intake in Hyper-LDL-Cholesterolemia

**Figure 3:**
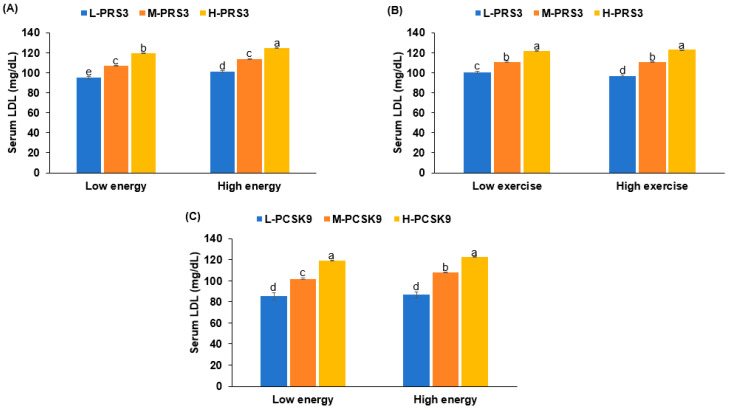
Serum LDL cholesterol according to PRS or PCSK9 rs151193009 and lifestyles interacting with PRS. (**A**): Serum LDL by PRS in low- and high-energy intake. (**B**): Serum LDL by PRS in low- and high-exercise. (**C**): Serum LDL by *PCSK9*_rs151193009 alleles in low- and high-energy intake. PRS was calculated by summing number of risk alleles of *PCSK9_rs151193009*, *CELSR2_rs11102967*, and *APOE_rs7412*. Covariates 1: BMI, residence area, gender, age, and education; covariates 2: parameters in covariates 1 plus energy intake, smoking status, physical activity, and alcohol intake. Mean ± SE is shown for each group after adjustment with covariates. Letters above bars represent statistically significant differences at *p* < 0.05 according to Tukey’s post hoc test.

The study revealed a significant interaction between the PRS and energy intake (*p* = 0.0016) (Table 5). Interestingly, as shown in Figure 3A, the genetic impact was less pronounced in participants with a high PRS than those with a low PRS, despite higher serum LDL-C concentrations in the high-PRS group. Additionally, significant interactions were observed between the PRS and physical activity (*p* = 0.0105) (Table 5). Figure 3B demonstrates that exercise could mitigate the genetic impact, but only in individuals with a low PRS.

The *PCSK9* genetic variant also interacted with energy intake (*p* = 0.0014), but not with exercise (Table 5). As illustrated in Figure 3C, a high energy intake exacerbated hyper-LDL-cholesterolemia in participants with a high PRS.

### 3.6. Molecular Interaction

**Figure 4:**
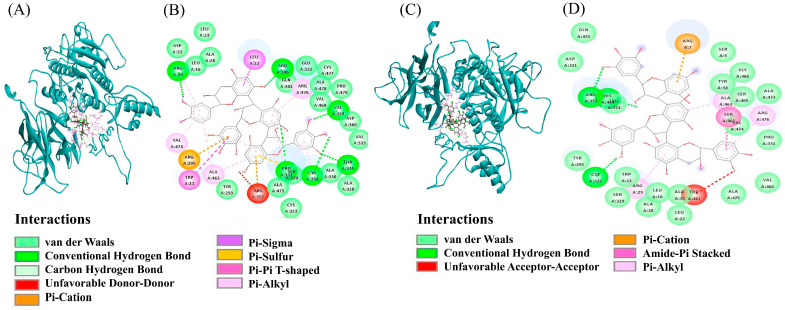
Molecular docking of WT (Arg93) and MT (93Cys) proteins generated from *PCSK9*_rs151193009 with prodelphinidin trimer GC-C-C gene. (**A**) Three-dimensional diagrammatic representation (ball-and-stick model) of prodelphinidin trimer binding to wild-type (WT, Arg93) PCSK9 protein. (**B**) Two-dimensional interaction map showing molecular docking interactions between WT PCSK9 protein and compound, illustrating types of stabilizing forces involved. Hydrogen bonds are depicted in dark green, while hydrophobic interactions are shown as arcs with radiating spokes. (**C**). Three-dimensional diagrammatic representation of compound bound to mutant (MT, 93Cys) PCSK9 protein. (**D**). Two-dimensional interaction map of MT PCSK9 protein complexed with compound, showing stabilizing interactions. Hydrophobic interactions are indicated by arcs with spokes, representing nonpolar contacts contributing to binding affinity. Additional interactions include van der Waals forces, alkyl, and carbon–hydrogen bonds. PCSK9, proprotein convertase subtilisin/kexin type 9.

The docking interactions of the selected active compounds with the wild-type (WT) and mutant (MT) forms of the PCSK9 (Arg93Cys) and APOE (Arg176Cys) proteins are presented in Tables S2 and S3, Figure 4, and Figures S3–S6, respectively. The molecular docking analysis showed that the binding energy between prodelphinidin trimer GC-C-C and PSCK9_WT was lower than that of PSCK9_MT. The binding energies of pelargonidin 3-O-[2-O-(6-(E)-feruloyl-beta-D-glucopyranosyl)-6-O-(E)-p-coumaroyl-beta-D-glucopyranoside] 5-O-(beta-D-glucopyranoside), 28-glucosyloleanolic acid 3-[arabinosyl-(1->2)-6-methylglucuronide], melongoside K, and momordin IIa to PSCK9_WT were higher than that of PSCK9_MT. It was found that *APOE* also has a low binding energy with the compounds mentioned earlier. The greater the number of hydrogen bonds between the compounds, the stronger and more stable the binding to PCSK9 and APOE. In Figure 4 and Figures S3–S6, dark green highlights indicate hydrogen bonds, pivotal to reducing the binding energy with PCSK9 and APOE. Hydrophobic interactions are depicted by arcs with spokes. The binding affinity of PCSK9 and APOE to the active compounds involves not just hydrogen bonds, but also van der Waals interactions, alkyl bonds, and carbon–hydrogen bonds. The screened compounds were selected according to the lowest energy ranking, and those with a threshold binding affinity of −10.0 kcal or greater were selected. Finally, five compounds were obtained, as mentioned in Tables S2 and S3, and Figure 4 and Figures S3–S6.

Setting up a control group and conducting parallel analysis can help to eliminate false positive results. This study selected eight drugs that can reduce the LDL concentration as ligands, and docked them with PCSK9 to rapidly explore potential inhibitors of PCSK9. The results showed that all eight drugs exhibited significant binding energies with PCSK9. The compounds in this study had lower binding energies than the positive control drug, and the difference was significant (Table S4).

**Figure 5:**
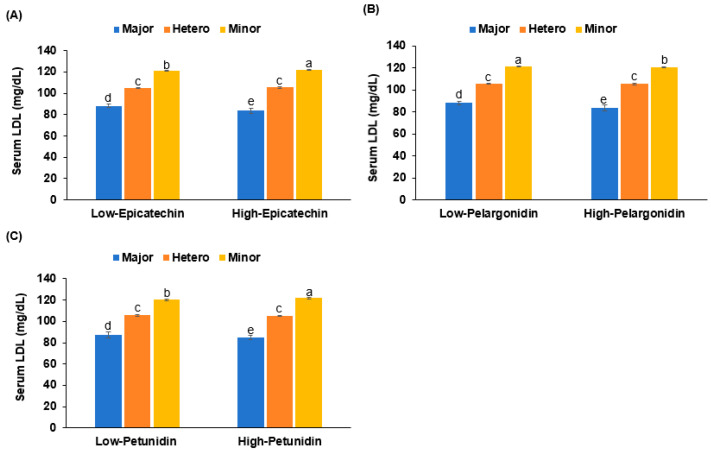
Serum LDL-C levels stratified by *PCSK9* rs151193009 genotype and dietary intake of epicatechin, pelargonidin, and petunidin—compounds structurally representative of key ligands that demonstrated low binding energy with PCSK9 wild-type protein in molecular docking analysis. (**A**) Serum LDL by *PCSK9*_rs151193009 alleles in low- and high-epicatechin. (**B**) Serum LDL by *PCSK9*_rs151193009 alleles in low- and high-pelargonidin. (**C**) Serum LDL by *PCSK9*_rs151193009 alleles in low- and high-petunidin. Significant gene–diet interactions were observed for both epicatechin and petunidin intake (*p* < 0.01), suggesting differential effects on LDL-C levels based on genetic variation. Mean ± SE is shown for each group after adjustment with covariates. Letters above bars indicate statistically significant differences (*p* < 0.05) according to Tukey’s post hoc test.

To further investigate the functional relevance of *PCSK9* rs151193009 variants, we examined whether dietary polyphenol intake modulated serum LDL-C levels differently depending on genotype. As shown in Figure 5, significant gene–diet interactions were observed. In participants with the wild type (major allele) of PCSK9, higher epicatechin intake—a polyphenol structurally similar to prodelphinidin—was associated with lower LDL-C levels. However, in individuals carrying the minor allele, higher epicatechin intake was paradoxically associated with increased LDL-C (*p* < 0.01). A similar interaction pattern was observed with petunidin intake, which decreased LDL-C in the major allele group, but increased it in the minor allele group. In contrast, pelargonidin intake led to a consistent decrease in LDL-C across all genotypes, although the effect was most pronounced in major allele carriers. These findings were consistent with our molecular docking data, where prodelphinidin and petunidin showed lower binding energy (stronger interaction) with the wild-type PCSK9 protein compared to the mutated form (Arg93Cys), whereas pelargonidin maintained relatively high binding affinity to both wild and mutant forms. The results suggest that specific dietary polyphenols may modulate PCSK9 function in a genotype-dependent manner, supporting a potential role for precision dietary strategies in managing LDL-C levels.

### 3.7. Molecular Dynamics (MD) Simulation

**Figure 6:**
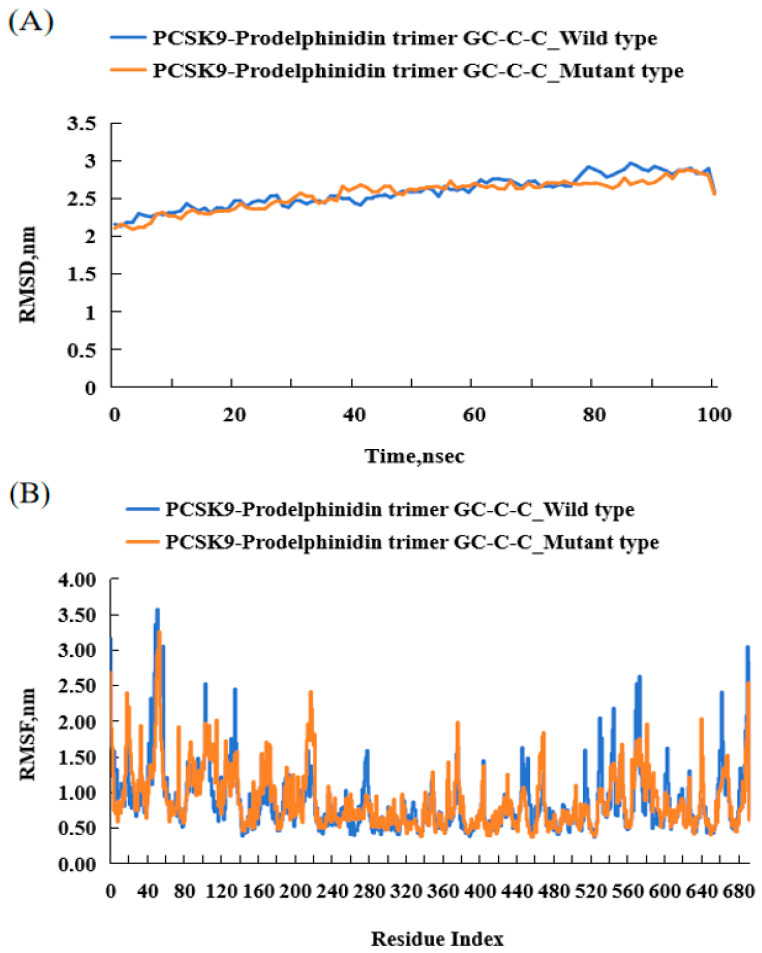
Molecular dynamics (MD) simulation of PCSK9_rs151193009 WT (Arg93) and MT (93Cys) proteins generated from *PCSK9*_rs151193009 with prodelphinidin trimer GC-C-C gene. (**A**). Root–mean–square deviation (RMSD) profiles showing structural stability of PCSK9 alone and PCSK9–compound complex over course of simulation. (**B**). Root–mean–square fluctuation (RMSF) values indicating residue-level flexibility of PCSK9 in absence and presence of compound. PCSK9, proprotein convertase subtilisin/kexin type 9; WT, wild type; MT, mutated type.

From MD simulation, we can understand the dynamics of the complex and the equilibrium of the protein structure. The average RMSD values of *PSCK9*_WT and MT of prodelphinidin trimer GC-C-C were 2.59 Å and 2.56 Å, respectively, while the average RMSD values of *PSCK9*_WT and MT of pelargonidin 3-O-[2-O-(6-(E)-feruloyl-beta-D-glucopyranosyl)-6-O-(E)-p-coumaroyl-beta-D-glucopyranoside] 5-O-(beta-D-glucopyranoside) were 2.51 Å and 2.44 Å, respectively (Figure 6). This finding may indicate that these substances have the most stable structure and retain the active site of the protein, and *PSCK9*_MT binding can stabilize the binding domain more than WT. In addition, the average RMSF values of *PSCK9*_WT and MT of prodelphinidin trimer GC-C-C were 0.88 Å and 0.90 Å, respectively, while the average RMSF values of *PSCK9*_WT and MT of pelargonidin 3-O-[2-O-(6-(E)-feruloyl-beta-D-glucopyranosyl)-6-O-(E)-p-coumaroyl-beta-D-glucopyranoside] 5-O-(beta-D-glucopyranoside) were 0.91 Å and 0.94 Å, respectively. This means that fluctuations increase with mutations (Figure 6 and Figure S7). The binding energy of PCSK9 with bioactive compounds was less than −9 kcal/mol, indicating stable ligand-receptor interactions in molecular docking frameworks.

## 4. Discussion

Our findings on the interactions between genetic factors, lifestyle choices, and LDL-cholesterol levels are particularly significant, as CVD remains the leading cause of mortality globally, surpassing cancer and other major health conditions [21]. Hyper-LDL-cholesterolemia is a well-established risk factor for atherosclerotic CVD, making it critical to understand its underlying mechanisms. We identified three key genes—CELSR2, PCSK9, and APOE—that play pivotal roles in lipid metabolism and may contribute to CVD development. *PCSK9*_rs151193009, *CELSR2*_rs11102967, and *APOE*_rs7412 are closely associated with disease risk in the current research population. However, there are significant differences in genetic structure among different populations, which is highly likely to affect the generalization performance of the model. Among these, PCSK9 stands out due to its role in regulating LDL-receptor (LDL-R) expression, making it a prime target for cholesterol-lowering therapies [22]. Located on chromosome 1p32, *PCSK9* mutations have been implicated in autosomal dominant hypercholesterolemia, particularly in French populations [23]. On the other hand, certain loss-of-function mutations in PCSK9 (such as Y142X and C679X) found in African Americans have been associated with lower cholesterol levels and a significantly reduced risk of CVD [24]. Furthermore, a meta-analysis by Qiu et al. [25] further supports the relevance of *PCSK9* in broader populations, showing that the rs11591147 T allele is significantly associated with lower total cholesterol (*p* < 0.0001) and LDL-C levels (*p* < 0.0001) and reduced CVD risk (OR: 0.77, 95% CI: 0.60 to 0.98, *p* = 0.031) in Caucasians. These findings highlight the broad applicability of PCSK9 research in mitigating CVD risk across diverse populations.

In our study, we carefully selected and adjusted for several confounding factors influencing serum LDL cholesterol levels. These factors included BMI, residential area, education level, gender, age, energy intake, alcohol intake, physical activity level, and smoking status. BMI was included as it directly relates to body fat content and lipid metabolism. The residential area and education level were considered as proxies for lifestyle, dietary habits, socioeconomic status, and healthcare access, all of which can indirectly affect LDL cholesterol. Gender and age were crucial factors due to physiological differences and age-related changes in lipid metabolism. Energy intake, rather than specific dietary patterns, was used as a confounding factor to account for the impact of overall calorie consumption on lipid levels [26]. Alcohol intake was included due to its effects on liver function and lipid metabolism, while physical activity level was considered for its known influence on cholesterol levels [27]. Smoking status was accounted for due to its impact on endothelial function and lipid metabolism [28]. Notably, we did not adjust for lipid-lowering medication use, as individuals taking such medications were categorized in the high-LDL group regardless of their current LDL levels, ensuring a comprehensive assessment of LDL-related risk. By adjusting for these factors, we aimed to isolate the effects of genetic variants and lifestyle interactions on LDL cholesterol levels, providing a more accurate assessment of their relationships in our study population.

*PCSK9*’s role in regulating blood cholesterol levels has been extensively studied. It interacts with LDL-Rs on liver cell surfaces, directing LDL to lysosomes for degradation [3,29]. Overexpression of *PCSK9* in mice leads to decreased hepatic LDL-R levels and elevated serum cholesterol, while *PCSK9* gene inactivation slows atherosclerosis progression [30,31]. *PCSK9* disrupts the normal LDL-R recycling process, reducing the liver’s ability to clear serum LDL-C and leading to its accumulation, thereby increasing CVD risk and accelerating atherosclerosis development [32]. *CELSR2*, also located on chromosome 1, encodes a transmembrane protein involved in cell adhesion and regulation of lipid metabolism [22]. Studies have shown that the loss of CELSR2 significantly reduces lipid accumulation in hepatocytes and impairs cell survival by inhibiting proliferation and promoting apoptosis [33]. Moreover, a strong correlation has been observed between *CELSR2* expression levels and LDL-C concentrations, making it a promising therapeutic target for lipid metabolism regulation [34,35].

*APOE*, located on chromosome 19, is primarily synthesized in the liver, and plays a crucial role in lipid metabolism [22]. Its functions include mediating the clearance of triglyceride-rich lipoproteins and their remnants, initiating reverse cholesterol transport to the liver, and distributing lipids to cells in the nervous system [36]. Different *APOE* isoforms have varying effects on LDL-C concentrations and atherosclerosis risk, significantly impacting cardiovascular health [22]. APOE exhibits a high affinity for LDL-R and mediates cholesterol uptake into the liver from the bloodstream [37]. Individuals with *APOE* mutations, such as the *APOE4* genetic variant, often display high serum LDL-C levels despite having a low BMI. This process involves LDL-R recycling to the cell surface, binding more circulating LDL particles, and further reducing serum LDL-C concentrations.

Our findings align with previous research, including a community cross-sectional study on Chinese adults that demonstrated an association between the *APOE*_rs7412 polymorphism and hyper-LDL-cholesterolemia [38]. Other studies have reported significant relationships between *APOE* genotypes and serum total cholesterol and LDL-C concentrations in healthy women, as well as associations between the *APOE*_rs7412 polymorphism and circulating serum total cholesterol and LDL-C levels [39,40]. The consistent findings across multiple studies, including ours, underscore the critical roles of *CELSR2*, *PCSK9*, and *APOE* in lipid metabolism. Considering all these factors, this model is likely to maintain good generalization ability in populations with similar genetic backgrounds, environmental factors, and research populations. However, in populations with significant genetic background differences or complex and variable environmental factors, the generalization ability of the model needs further validation and optimization.

In this study, the 3-SNP model included *PCSK9_rs151193009*, *CELSR2_rs11102967*, and *APOE_rs7412*. The PRS of the 3-SNP model positively correlated with hyper-LDL-cholesterolemia. Interactions between the PRS, high energy intake, and exercise in individuals with hyper-LDL-cholesterolemia were also observed. High energy intake exacerbated the high-PRS effect, while exercise attenuated it. Notably, *PCSK9*_rs151193009 interacted with energy intake, amplifying the risk allele’s effects on hyper-LDL-cholesterolemia. These findings suggest that individuals with a high PRS and the *PCSK9*_rs151193009 risk allele should consume less energy than their estimated requirement. Moreover, exercise appears to help mitigate the PRS impact. Previous research has shown that a WSD dietary pattern, characterized by increased energy intake, exacerbates the genetic effects of hyper-LDL-cholesterolemia [15]. Our results further support the notion that in genetically predisposed individuals, hyper-LDL-cholesterolemia could be closely associated with energy balance, highlighting the complex interplay between genetic predisposition and lifestyles in influencing serum LDL levels, emphasizing the potential for personalized interventions in managing CVD risk.

To deepen our understanding of gene–diet interactions and their mechanistic implications, we employed in silico molecular docking to predict how dietary polyphenols may interact with PCSK9 and related proteins. This computational approach complements our epidemiological findings and offers insight into how specific compounds could modulate lipid metabolism in a genotype-dependent manner. Our analysis focused on the *PCSK9* missense mutation Arg93Cys (rs151193009), which was significantly associated with serum LDL-C levels. Using AutoDock, we simulated the binding interactions of structurally diverse dietary compounds with both wild-type and mutant forms of *PCSK9* and *APOE*. Notably, prodelphinidin trimer GC-C-C and glycated pelargonidin demonstrated substantially lower binding energies across both protein forms, suggesting a strong potential to influence their activity. These computational results are supported by our observational data, where related polyphenols such as epicatechin and petunidin exhibited genotype-specific effects on serum LDL-C. Interestingly, certain compounds—specifically glycated glucosylorenolenic acid, melongoside K, and momordin IIa—showed reduced binding energy exclusively with the mutant PCSK9 (Cys93), indicating potential selective efficacy for individuals carrying this rare variant. This supports the concept of genetically tailored dietary interventions. From a pharmacological standpoint, docking energies between –9 and –12 kcal/mol, as observed in our study, are generally considered indicative of strong and stable ligand–receptor interactions in computational drug screening. These values are comparable to binding affinities of small-molecule inhibitors in early-phase drug discovery. However, it is important to emphasize that docking scores primarily reflect binding potential, and do not capture downstream biological effects such as compound solubility, metabolic stability, cellular permeability, or systemic bioavailability. As such, while these findings are promising, further validation through functional assays and pharmacokinetic profiling is required before clinical translation.

Conversely, *APOE* interactions were less influenced by genotype, as reflected in their relatively high and stable binding energies. These findings suggest that the *PCSK9* rs151193009 variant may offer a more responsive target for dietary modulation. While cell-based validation assays such as LDL uptake or PCSK9 secretion remain valuable, our current study demonstrates the utility of in silico screening to prioritize candidate compounds and pathways. Future work will incorporate functional validation once experimental conditions are optimized. These findings lay a foundation for personalized nutrition strategies and underscore the promise of PCSK9 as a functional food or therapeutic target.

This study has several limitations that warrant consideration. First, the cross-sectional design limits causal inference regarding the relationship between polygenic risk scores (PRSs), PCSK9 variants, lifestyle factors, and serum LDL-C concentrations. Future longitudinal studies are needed to validate these associations over time and assess their impact on cardiovascular outcomes. Second, while our molecular docking analyses provided mechanistic insights into potential ligand–PCSK9 interactions, we did not perform site-directed mutagenesis or cell-based functional assays, such as co-immunoprecipitation and Western blotting, to directly validate binding site specificity. These experiments are crucial for confirming the predicted molecular interactions, and should be prioritized in future work. Third, the functional effects of candidate bioactive compounds—including prodelphinidin trimer GC-C-C, glycated pelargonidin, glycated glucosylorenolenic acid, melongoside K, and momordin IIa—remain to be experimentally validated. Although our in silico models suggest promising binding affinities, future in vitro assays using hepatocyte cell lines and in vivo animal models are required to assess their physiological impact on LDL-C regulation, bioavailability, metabolism, and safety. At present, these compounds remain at the early stage of basic research, and there is a considerable translational gap before they can be considered for clinical or nutritional applications. Large-scale clinical trials will ultimately be needed to confirm their efficacy and safety in humans. Fourth, our study did not include epigenetic markers, such as DNA methylation or histone modifications, which may interact with genetic variants to influence gene expression and lipid metabolism. Integrating epigenomic data with PRS models could provide a more comprehensive understanding of gene regulation and should be explored in future multi-omics studies. Finally, our PRS model, while biologically driven, has methodological constraints. It was constructed using three GMDR-identified SNPs (*PCSK9* rs151193009, *CELSR2* rs11102967, *APOE* rs7412), selected for their known epistatic interactions and functional relevance in cholesterol metabolism pathways [41]. Although this approach enhanced interpretability and avoided overfitting, it does not capture the full polygenic architecture typically addressed by contemporary genome-wide PRS models that include hundreds of SNPs. Several genome-wide significant SNPs were excluded due to a lack of functional annotation or absence of interaction with rs151193009. Future work should apply weighted PRS approaches using large biobank-scale datasets (e.g., UK Biobank) to improve the resolution and predictive power of the genetic signature. Despite these limitations, our study demonstrates the potential of integrating genetic data, lifestyle factors, and in silico modeling to identify novel targets for LDL-C modulation. The approach offers a promising framework for future precision nutrition and therapeutic discovery efforts targeting the PCSK9–LDLR pathway.

The novelty of the present study was that these selected compounds showed promise in disrupting the interaction between *PCSK9* and LDL-R, which could play a significant role in CVD management. To target this interaction, these natural compounds might offer a new approach to regulating LDL-C levels, potentially leading to more effective and possibly better-tolerated treatments for hypercholesterolemia. Furthermore, our study demonstrates the value of combining genetic analysis with in silico modeling to identify potential therapeutic targets and compounds. This approach could be applied to other complex diseases, potentially accelerating drug discovery and development.

## 5. Conclusions

A 3-SNP PRS model (*PCSK9*_rs151193009, *CELSR2*_rs11102967, and *APOE*_rs7412) outperformed single SNP analysis in predicting hyper-LDL-cholesterolemia. The model’s predictive power was strong when combined with lifestyle factors, notably high energy intake (OR: 3.678, 95% CI: 2.460–5.499) and low physical activity (OR: 4.094, 95% CI: 2.822–5.940). These findings suggest that individuals with high genetic risk might benefit significantly from lifestyle modifications, especially low-energy diets and increased physical activity. Our molecular docking analysis identified potential bioactive compounds, including the prodelphinidin trimer GC-C-C, that may modulate APOE (Arg176Cys) and PCSK9 (Arg93Cys) activities. While these in silico results are promising, they require further experimental validation. This study lays the groundwork for personalized hyper-LDL cholesterolemia management strategies that integrate genetic risk assessment, lifestyle factors, and targeted interventions. Future research should aim to validate these findings across diverse populations and investigate the clinical applicability of the identified compounds.

## Figures and Tables

**Table 1:** Demographic and metabolic characteristics of the participants according to serum LDL concentration.

	Men (*n* = 20,293)		Women (*n* = 38,408)	
	Low-LDL (*n* = 17,845)	High-LDL (*n* = 2448)	Low-LDL (*n* = 31,890)	High-LDL (*n* = 6518)
Age (years)	56.2 ± 0.06 ^b^	57.3 ± 0.15 ^a^	51.9 ± 0.04 ^d^	55.3 ± 0.09 ^c^***^+++###^
Education (N, %)				
≤Middle school	1582 (14.0)	171 (12.1) ^†^	5340 (21.4)	1398 (25.7) ^†††^
High school	8361 (75.6)	1073 (76.0)	18,085 (72.5)	3786 (69.7)
≥Collage	1123 (10.2)	168 (11.9)	1534 (6.15)	248 (4.57)
Income (dollar/month)				
≤2000	1442 (8.49)	164 (7.07) ^††^	3289 (11.0)	883 (14.4) ^†††^
2000–4000	7273 (42.8)	934 (40.3)	13,058 (43.5)	2927 (47.8)
>4000	8278 (48.7)	1222 (52.7)	13,661 (45.5)	2309 (37.7)
BMI (kg/m^2^)	24.3 ± 0.02 ^b^	25 ± 0.06 ^a^	23.5 ± 0.02 ^c^	24.3 ± 0.04 ^b^***^+++^
Waist circumference (cm)	84.5 ± 0.04 ^b^	85 ± 0.1 ^a^	78.6 ± 0.03 ^d^	79.2 ± 0.06 ^c^***^+++^
SMI (kg/m)	7.14 ± 0.005 ^a^	7.13 ± 0.011 ^a^	6.14 ± 0.003 ^b^	6.03 ± 0.007 ^c^***^+++###^
Fat mass (%)	22.7 ± 0.01 ^d^	23 ± 0.02 ^c^	31.2 ± 0.01 ^b^	31.8 ± 0.01 ^a^***^+++###^
Serum glucose (mg/dL)	98.1 ± 0.19 ^b^	100.5 ± 0.42 ^a^	92.7 ± 0.13 ^d^	96.6 ± 0.25 ^c^***^+++##^
HbA1c (%)	5.71 ± 0.01 ^b^	5.9 ± 0.02 ^a^	5.65 ± 0.01 ^c^	5.9 ± 0.01 ^a^*^+++#^
Insulin resistance (yes, %)	1943 (10.9)	369 (15.1) ^†††^	1634 (5.12)	658 (10.1) ^†††^
Total-C (mg/dL)	186 ± 0.31 ^d^	222 ± 0.69 ^b^	194 ± 0.21 ^c^	237 ± 0.42 ^a^***^+++###^
HDL-C (mg/dL)	49.3 ± 0.12 ^d^	50.8 ± 0.27 ^c^	55.9 ± 0.08 ^b^	56.5 ± 0.16 ^a^**^+++##^
Triglyceride (mg/dL)	134 ± 0.78 ^a^	135 ± 1.76 ^a^	118 ± 0.53 ^c^	128 ± 1.06 ^b^***^+++###^
LDL-C (mg/dL)	110 ± 0.28 ^d^	144 ± 0.63 ^b^	114 ± 0.19 ^c^	155 ± 0.38 ^a^***^+++###^
Lipid-lowering medication (N, %)	0 (0)	941(40.5) ^†††^	0 (0)	1788 (30.7) ^†††^
LDL-C on lipid-lowering medication (mg/dL)		97.4 ± 1.45		105 ± 1.01 ^††^
LDL-C on no lipid-lowering medication (mg/dL)		177 ± 0.70		179 ± 0.41
SBP (mmHg)	126 ± 0.13 ^a^	126 ± 0.3 ^a^	120 ± 0.09 ^c^	123 ± 0.18 ^b^***^+++###^
DBP (mmHg)	78.3 ± 0.09 ^a^	78.5 ± 0.2 ^a^	74.1 ± 0.06 ^c^	75.4 ± 0.12 ^b^

Values represent adjusted means and standard errors after adjusting for covariates of age, gender, education, residence area, body mass index (BMI), energy intake, smoking, alcohol intake, and exercise. An independent variable was eliminated from the covariates. HbA1c, hemoglobin A1c; Total-C, total cholesterol; LDL-C, low-density lipoprotein cholesterol; HDL-C, high-density lipoprotein cholesterol; SBP, systolic blood pressure; DBP, diastolic blood pressure; SMI, skeletal muscle index. Low- and high-LDL groups were categorized with the cutoff of 130 mg/dL serum LDL-cholesterol concentration, and the high-LDL group included participants taking lipid-lowering medication. ^+++^ Significant differences with serum LDL by two-way ANOVA at *p* < 0.001. * Significant differences with genders by two-way ANOVA at *p* < 0.05, ** at *p* < 0.01, and *** at *p* < 0.001. ^#^ Significant interaction between gender and serum LDL concentration by two-way ANOVA at *p* < 0.05, ^##^ at *p* < 0.01, and ^###^ at *p* < 0.001. ^a,b,c,d^ Different letters indicate significant differences among the groups in Tukey’s test at *p* < 0.05. ^†^ Significantly different from the low-LDL group in each gender by χ^2^ test at *p* < 0.05, ^††^ at *p* < 0.01, and ^†††^ at *p* < 0.001.

**Table 2:** General characteristics of the study population according to serum LDL concentration.

	Men (*n* = 20,293)		Women (*n* = 38,408)	
	Low-LDL (*n* = 17,845)	High-LDL (*n* = 2448)	Low-LDL (*n* = 31,890)	High-LDL (*n* = 6518)
Energy intake (EER %)	90 ± 0.29 ^c^	89.8 ± 0.66 ^c^	99.1 ± 0.2 ^b^	101 ± 0.4 ^a^***^+##^
Fiber (g/day)	14.5 ± 0.07 ^b^	14.1 ± 0.17 ^b^	14.8 ± 0.05 ^a^	15 ± 0.1 ^a^***^##^
Ca (mg/day)	391 ± 1.84 ^c^	396 ± 4.17 ^c^	467 ± 1.25 ^b^	483 ± 2.52 ^a^***^+++#^
Na (mg/day)	2428 ± 11 ^a^	2358 ± 24.9 ^b^	2433 ± 7.4 ^a^	2444 ± 15 ^a^*^+##^
Vitamin C (mg/day)	92.6 ± 0.53 ^b^	91.8 ± 1.21 ^b^	112.4 ± 0.36 ^a^	112.8 ± 0.73 ^a^***
Vitamin D (ug/day)	5.3 ± 0.05 ^c^	5.6 ± 0.11 ^c^	6.9 ± 0.03 ^b^	7.2 ± 0.06 ^a^***^+++^
DII	−18.9 ± 0.13	−18.5 ± 0.3	−20.4 ± 0.09	−21.1 ± 0.18***^##^
Flavonoids (mg/day)	31.4 ± 0.28 ^b^	31.6 ± 0.63 ^b^	42.2 ± 0.19 ^a^	41.8 ± 0.38 ^a^***
KBD (Yes, N, %)	7148 (40.6)	953 (38.9)	9534 (29.9)	1934 (29.7)
PBD (Yes, N, %)	14,230 (20.3)	583 (23.8) ^†^	12,751 (40.0)	2627 (40.3)
WSD (Yes, N, %)	9096 (51.0)	1335 (54.5) ^++^	11,078(34.7)	2043 (31.4) ^†††^
RMD (Yes, N, %)	5669 (31.8)	796 (32.5)	11,015(34.5)	2091(32.1) ^†††^
Alcohol (g/week)	28 ± 0.42 ^a^	23.7 ± 0.95 ^b^	9.9 ± 0.28 ^c^	7.8 ± 0.58 ^d^***^+++#^
Exercise (Yes, N, %)	16,522 (52.0)	3502 (53.9) ^†††^	10,446 (58.7)	1506 (61.8) ^††^
Former smoking (Yes, N, %)	7543 (43.0)	1146 (47.6) ^†††^	376 (1.20)	79 (1.23)
Smoking (Yes, N, %)	4938 (28.1)	643 (26.7)	611 (1.94)	126 (1.96)

Values represent adjusted means ± standard errors or number (N) and percentage. The covariates included age, gender, education, residence area, body mass index, metabolic syndrome, energy intake, smoking, alcohol intake, fat intake, physical activity, and any medication for inflammatory diseases. An independent variable was eliminated from the covariates. Low- and high-LDL groups were categorized with the cutoff of 130 mg/dL serum LDL cholesterol concentration, and the high-LDL group included participants taking lipid-lowering medication. DII, dietary inflammatory index; KBD, Korean balanced diet; PBD, plant-based diet; WSD, Western-style diet; RMD, rice-main diet. ^+^ Significant differences in serum LDL concentrations by two-way ANOVA at *p* < 0.05, ^++^ at *p* < 0.01, and ^+++^ at *p* < 0.001. * Significant differences with genders by two-way ANOVA at *p* < 0.05, *** at *p* < 0.001. # Significant interaction between gender and serum LDL concentration by two-way ANOVA at *p* < 0.05 and ## at *p* < 0.01. ^†^ Significantly different from the low-LDL group in each gender by χ^2^ test at *p* < 0.01, ^††^ at *p* < 0.01 and ^†††^ at *p* < 0.001. ^a,b,c,d^ Different letters indicate significant differences among the groups in Tukey’s test at *p* < 0.05.

**Table 3:** The characteristics of the three SNPs selected by the generalized multifactor dimensionality reduction analysis.

CHR	SNP	BP	A1	A2	MAF	HWE	OR	SE	P	OR	SE	P1	Location	Gene Names
1	rs151193009	55509585	T	C	0.015	0.208	0.330	0.132	5.97 × 10^−17^	0.158	0.453	4.82 × 10^−5^	missense (Arg93Cys)	*PCSK9*
1	rs11102967	109817245	C	A	0.060	0.286	0.689	0.049	2.66 × 10^−14^	0.692	0.093	6.90 × 10^−5^	3′ UTR	*CELSR2*
19	rs7412	45412079	T	C	0.065	0.29	0.324	0.064	1.05 × 10^−68^	0.268	0.164	8.63 × 10^−16^	missense (arg176cys)	*APOE*

*PCSK9*, Proprotein Convertase Subtilisin/Kexin Type 9; *CELSR2*, Cadherin EGF LAG Seven-Pass G-Type Receptor 2; *APOE*, Apolipoprotein E; CHR, chromosome; SNP, single-nucleotide polymorphism; BP, position of SNP in chromosome. A1, minor allele; A2, major allele; MAF, minor allele frequency; HWE, *p* value for Hardy–Weinberg equilibrium analysis. OR, odds ratio for hyper-LDL-cholesterolemia in reference to major allele; P, *p* value for OR adjusted for BMI, gender, age, education, residence area, energy intake, alcohol intake, physical activity, and smoke in the city cohort; P1. *p* value for OR adjusted for covariates in Ansan/Ansung cohort and rural cohort.

**Table 4:** Adjusted odds ratios for metabolic parameters according to the PRS of model 3 for hyper-LDL-cholesterolemia.

	Low-PRS (*n* = 14,420)	Medium-PRS (*n* = 21,641)	High-PRS (*n* = 4201)	Adjusted OR and 95% CI
LDL-C (mg/dL) ^1^	99 ± 0.82 ^c^	110.6 ± 0.28 ^b^	122.3 ± 0.15 ^a^***	3.451 (2.771–4.298)
BMI (kg/m^2^) ^2^	23.7 ± 0.07	23.9 ± 0.03	23.9 ± 0.01	1.068 (0.953–1.198)
Waist circumference (cm) ^3^	80.6 ± 0.2	80.8 ± 0.07	80.7 ± 0.04	1.077 (0.943–1.23)
Total cholesterol (mg/dL) ^4^	181 ± 0.90 ^c^	190 ± 0.31 ^b^	200 ± 0.17 ^a^***	2.452 (2.084–2.884)
HDL-C (mg/dL) ^5^	55.8 ± 0.32 ^a^	54.7 ± 0.11 ^b^	53.5 ± 0.06 ^c^***	1.439 (1.267–1.634)
TG (mg/dL) ^6^	131 ± 1.73 ^a^	123 ± 0.60 ^b^	120 ± 0.33 ^c^***	0.818 (0.728–0.92)
SMI (kg/m) ^7^	6.46 ± 0.01	6.46 ± 0	6.46 ± 0	0.997 (0.882–1.127)
Fat mass (%) ^8^	28.3 ± 0.03	28.4 ± 0.01	28.4 ± 0	1.116 (0.997–1.25)
Serum glucose (mg/dL) ^9^	95.7 ± 0.5	95 ± 0.17	95 ± 0.09	0.946 (0.809–1.105)
HbA1c (%) ^10^	5.7 ± 0.02	5.7 ± 0.01	5.7 ± 0	1.045 (0.803–1.359)
Insulin resistance (yes, %)	383 (7.29)	2840 (7.70)	1241 (7.72)	1.036 (0.841–1.277)
SBP (mmHg) ^11^	122 ± 0.36	122 ± 0.12	122 ± 0.07	1.038 (0.924–1.166)
DBP (mmHg) ^12^	76.1 ± 0.24	75.7 ± 0.08	75.7 ± 0.05	0.96 (0.802–1.15)

Values represent mean ± SE or adjusted odd ratios (ORs) and 95% confidence intervals (CsI) after adjustment with covariates. Gene–gene interaction model with 3 SNPs included *APOE*_rs7412, *CELSR2*_rs11102967, and *PCSK9*_rs151193009. Low-PRS, medium-PRS, and high-PRS were divided into 0–2, 3–4, and >4 risk alleles for 3-SNP model, respectively. Cutoffs of each variable for logistic regression: ^1^ 130 mg/dl; ^2^ 25 kg/dl; ^3^ 90 cm for M and 85 cm for women; ^4^ 230 mg/dL; ^5^ 40 mg/dL for M and 50 mg/dL for W; ^6^ 150 mg/dL; ^7^ 7.2 kg/m; ^8^ 25% for M and 30% for W; ^9^ 110 mg/dL; ^10^ 6.5%; ^11^ 120 mmHg; ^12^ 80 mmHg. *** Significantly different from low-PRS group in logistic regression analysis at *p* < 0.001. Superscript letters indicate statistically significant differences among groups at *p* < 0.05 according to Tukey’s post hoc test.

**Table 5:** Adjusted odds ratios for hyper-LDL-cholesterolemia by the risk of *PCSK9* rs151193009 after accounting for covariates, based on lifestyle patterns.

PRS3	Low-PRS (*n* = 14,420)	Medium-PRS (*n* = 21,641)	High-PRS (*n* = 4201)	Gene–Nutrient Interaction *p* Value
Low energy ^1^ High energy	1	1.761 (1.317–2.356) 1.801 (1.191–2.724)	3.361 (2.534–4.458) 3.678 (2.460–5.499)	0.0016
Low exercise ^2^ High exercise	1	1.995 (1.361–2.924) 1.694 (1.243–2.308)	4.094 (2.822–5.940) 3.149 (2.331–4.252)	0.0105
PCSK9	Non-risk (*n* = 51,346)	Heterozygotes (*n* = 7126)	Risk (*n* = 229)	Gene-nutrient interaction *p* value
Low energy High energy	1	0.999 (0.996–1.001) 0.995 (0.991–0.998)	2.504 (2.216–2.830) 2.716 (2.292–3.217)	0.0014
Low exercise High exercise	1	0.996 (0.993–0.999) 0.998 (0.995–1.001)	2.659 (2.286–3.093) 2.471 (2.169–2.815)	0.1128

Values are adjusted odds ratios and 95% confidence intervals. Adjusted odds ratios for hyper-LDL-cholesterolemia by polygenetic risk scores of best model (PRS) for gene–gene interaction, after covariate adjustments according to lifestyle patterns. Gene–gene interaction model with 3 SNPs included *APOE*_rs7412, *CELSR2*_rs11102967, and *PCSK9*_rs151193009. Low-PRS, medium-PRS, and high-PRS were divided into 0–2, 3–4, and >4 risk alleles for 3-SNP GMDR model, respectively. When analyzing adjusted ORs, reference cutoff points were as follows: ^1^ <70 energy percentage (En%); ^2^ <150 min/week moderate physical activity score. In building regression model, interaction model was employed to investigate relationship between PRS and lifestyle factors.

## Data Availability

All data are included in the manuscript.

## Supplementary Materials

The following supporting information can be downloaded at https://www.mdpi.com/article/10.3390/nu17091411/s1: Supplementary Table S1. Generalized multifactor dimensionality reduction (GMDR) of SNP–SNP interaction of genes linked to serum LDL concentration. Supplementary Table S2. Chemical and clinical characterization of foods subjected to virtual screening for *PCSK9* major protease. The minimum binding energy of the compound to *PCSK9*. Supplementary Table S3. Chemical and clinical characterization of foods subjected to virtual screening for *APOE* major protease. The minimum binding energy of the compound to *APOE*. Supplementary Table S4. Results of LDL lowering drug PCSK9 molecular docking based on positive control. Supplementary Figure S1. Genetic variant distribution for serum LDL concentration risk in a genome-wide association study. Supplementary Figure S2A. Adjusted odd ratios (OR) and 95% confidence intervals (CI) of hyper-LDL-cholesterolemia (excluding the participants with lipid-lowering medication) with genetic variants and their polygenic risk score (PRS). Supplementary Figure S2B. Adjusted odd ratios (OR) and 95% confidence intervals (CI) of hyper-LDL-cholesterolemia (High-LDL defined as ≥130 mg/dL) with genetic variants and their polygenic risk score (PRS). Supplementary Figure S3. Molecular docking of *PCSK9*_rs151193009 (Arg93) and MT (93Cys) with a bioactive compound for Pelargonidin 3-O-[2-O-(6-(E)-feruloyl-beta-D-glucopyranosyl)-6-O-(E)-p-coumaroyl-beta-D-glucopyranoside] 5-O-(beta-D-glucopyranoside). Supplementary Figure S4. Molecular docking of *PCSK9*_rs151193009 (Arg93) and MT (93Cys) with a bioactive compound for 28-Glucosyloleanolic acid 3-[arabinosyl-(1->2)-6-methylglucuronide]. Supplementary Figure S5. Molecular docking of *PCSK9*_rs151193009 (Arg93) and MT (93Cys) with a bioactive compound for Melongoside K. Supplementary Figure S6. Molecular docking of *PCSK9*_rs151193009 (Arg93) and MT (93Cys) with a bioactive compound for Momordin IIa. Supplementary Figure S7. Molecular dynamics (MD) simulation of PCSK9_rs151193009 (Arg93) and MT (93Cys) and Pelargonidin 3-O-[2-O-(6-(E)-feruloyl-beta-D-glucopyranosyl)-6-O-(E)-p-coumaroyl-beta-D-glucopyranoside] 5-O-(beta-D-glucopyranoside).
