# Supplementary material for: Genetic Variants, Bioactive Compounds, and PCSK9 Inhibitors in Hyper-LDL-Cholesterolemia: A GWAS and In Silico Study on Cardiovascular Disease Risk
Source: Nutrients. 2025 Apr 23;17(9):1411. doi: 10.3390/nu17091411 (PMC12073296; doi:10.3390/nu17091411)
Supplement: Supplementary file 1 [file nutrients-17-01411-s001.zip › nutrients-3585701-supplementary.pdf]

Table S1. Generalized multifactor dimensionality reduction (GMDR) of genetic variant-genetic variant interaction of genes related to Serum LDL concentration.

GMDR	Adjusted for SEX, AGE, BMI, EDU, INCOME, AREA)				Adjusted for SEX, AGE, BMI, EDU, ALCOHOL, SMOKE, EXER, AREA, Energy intake)			
Model	TRBA	TEBA	P value	CVC	TRBA	TEBA	P value	CVC
*APOE*_rs7412	0.5431	0.5431	0.001	10	0.5431	0.5431	0.001	10
*CELSR2*_rs11102967 plus model 1	0.5542	0.5542	0.001	10	0.5542	0.5542	0.001	10
*PCSK9*_rs151193009 plus model 2	0.5614	0.5615	0.001	10	0.5614	0.5615	0.001	10
*TXNL4B*_rs3794694, *ANKDD1B*_rs9332464, plus model 2	0.5642	0.5608	0.001	9	0.5642	0.5608	0.001	9
*TXNL4B*_rs217184, *APOB*_rs13306206 plus model 3	0.5695	0.5664	0.001	10	0.5695	0.5664	0.001	10
*APOC1*_ rs12721051 plus model 5	0.5734	0.5686	0.001	10	0.5734	0.5686	0.001	10
*APOC1*_ rs12721051, HMGCR_ rs2878419, *LDLR*_rs688, plus model 4	0.5777	0.5579	0.001	8	0.5777	0.5579	0.001	8
*NECTIN2*_rs283813, plus model 7	0.5846	0.5612	0.001	10	0.5846	0.5612	0.001	10
*PCSK9*_rs151193009, plus model 8	0.5899	0.5621	0.001	10	0.5899	0.5621	0.001	10
*TOMM40*_rs1160983, plus model 9	0.5927	0.5598	0.001	10	0.5927	0.5598	0.001	10

BMI, body mass index; EDU, education; AREA, residence area.

Table S2. Chemical and clinical characterization of foods subjected to virtual screening for PCSK9 major protease. The minimum binding energy of the compound to PCSK9

Generic name	Effective food	Residues involved in hydrogen bonding		Residues involved in hydrophobic interactions		Wild type	Mutant type
		Wild type	Mutant type	Wild type	Mutant type	Docking energy, ΔG (kcal mol−1)	
Prodelphinidin trimer GC-C-C	Malt	Arg29,Arg306,Val359,Cys358,Thr335,Pro331	Arg357,His464,Cys323	Arg29,Val474,Ala463,Arg29,Trp11,Arg357,Arg476,Leu22,Pro331,Cys358	Arg29,Trp461,Arg476,Ser462,Ala463,Arg7	-11.9	-9.8
Pelargonidin 3-O-[2-O-(6-(E)-feruloyl-beta-D-glucopyranosyl)-6-O-(E)-p-coumaroyl-beta-D-glucopyranoside] 5-O-(beta-D-glucopyranoside)	Radish	Gln302,Ala28,Arg29,Leu23,Arg357,Ser329,Cys323,Arg295	Thr459,Arg458,Arg306,Tyr293,Ser329,Pro331	Arg306,Leu22,Glu332,Ala28,Leu23,Gly504,Ala26,Pro331,Arg458,Cys358,Arg476	Leu440,Pro438,Ile416,Arg458,Arg476,Leu22,Val474,Ala28,Ala463,Tyr293,Arg357,Leu21,Ala299,Leu15,Ala478,	-11.3	-12.4
Petunidin 3-O-[6-O-(4-O-(4-O-(beta-D-glucopyranosyl)-feruloyl)-alpha-L-rhamnopyranosyl)-beta-D-glucopyranoside]- 5-O-[beta-D-glucopyranoside] 1092	Blueberries, red grapes	Arg7,GLN302,ASN439,ALA463,Val474,Arg458,Arg476,	Ala28,Arg29,Ala30,Arg357,His464, Trp461	Tyr293, Arg306,Cys323,Leu324,Glu332,Val333,Val359,Thr437,Thr459,Thr461,Ser462,	Ser5,TRP11,Leu16,Leu22,Gln31,Tyr38,Glu40,Cys323,Tyr293,Arg295,Ser329	-11.6	-8.5

				Val650, Ser			
28-Glucosyloleanolic acid 3-[arabinosyl-(1->2)-6-methylglucuronide]	cardoon	Leu43,Leu18	Arg458,Asp360, Val359,Ala330,	Leu43,Leu17,Leu18,Leu49,Leu20,Leu21,Pro14, Glu84,Lys83,Asp141,Leu82,Tyr142,Gly121,Leu119,Val81,Val140,Ile143,Thr63, Val296,Pro120, His139,Val80	Cys358,Arg476, Ala463,Leu16, Val474,Ser465, Ala30,Thr472,Ala471,Arg29,Trp11,Pro331,	-7.2	-11.6
Melongoside K	aubergine	Asp33,Leu16, Asp37,Glu34, Leu18,Glu49, Gly36,Gly27, Ala26,Leu41,	Ala28,Val474,Arg476,Trp461,Arg357,Ser329	Leu17,Leu20,Leu19,Leu18,Glu49,Pro25,Asp35, Gly36,Gly27,Glu34,Val42,Ala26,Ala44,Leu41,Leu43,Ser47,Leu13	Pro331,Ala475, Ala463,Arg458, Cys323,Leu324, Arg29,Tyr293	-6	-12
Momordin IIa	Momordica cochinchinensis	Leu17,Glu34, Glu40	Thr472,Ser465, Gln31,Ala30,Arg29,Val359,Val333,Arg458,Arg357,Trp461	Ala28,Leu15,Leu22,Leu23,Leu21,Leu16,Leu17, Leu18,Leu19,Leu20,Leu41,Leu43,Ala26,Gly24, Gly27,Gly36,Pro25,Glu34,Asp35,Val42,Ala44,S	Ser465,Gly466, Ala30,Gln31,Arg29,Val474,His464,Ala463,Arg476,Pro331,Asp360,Arg458,Trp461	-7	-12

er47,Pro14,Leu1 3

Table S3. Chemical and clinical characterization of foods subjected to virtual screening for APOE major protease. The minimum binding energy of the compound to APOE

Generic name	Effective food	Residues involved in hydrogen bonding		Residues involved in hydrophobic interactions		Wild type	Mutant type
		Wild type	Mutant type	Wild type	Mutant type	Docking energy, ΔG (kcal mol−1)	
Prodelphinidin trimer GC-C-C	Malt	Ala120,Arg198,Gln205,Gly206,Ala259,Arg258,Gln135,Glu262,Gln266	Ala120,Arg198,Gln205,Gly206,Ala259,Arg258,Gln135,Glu262,Gln266	Ala124,Gly131,Glu256,Arg258,Ala259,Asp128	Ala120,Arg198,Gln205,Gly206,Ala259,Arg258,Gln135,Glu262,Gln266	-7.7	-7.7
Pelargonidin 3-O-[2-O-(6-(E)-feruloyl-beta-D-glucopyranosyl)-6-O-(E)-p-coumaroyl-beta-D-glucopyranoside] 5-O-(beta-D-glucopyranoside)	radish	Lys2,Glu29,Thr26,Gln34,Glu37	Glu37,Gln34,Lys2,Glu29,Thr26	Lys2,Trp5,Pro30,Arg33,Asp53,Glu37,Val10	Lys2,Glu37,Val10,Trp5,Pro30,Arg33,Asp53,	-8.3	-8.3
28-Glucosyloleanolic acid 3-[arabinosyl-(1->2)-6-methylglucuronide]	cardoon	Arg198,Gln266,Arg258,Glu262	Arg198,Gln266,Arg258,Glu262	Leu202,Gln205,Ala259,Asp128,Glu127,Gly	Leu202,Gln205,Ala259,Asp128,Glu127,Gly131	-8	-8.1

				131			
Melongoside K	aubergine	Gln266,Glu262	Gln266,Glu262	Arg258,Ala259,Val134,ALA211,Cys130,Glu127	Arg258,Ala259,Val134,ALA211,Cys130,Glu127	-7.8	-7.7
Momordin IIa	Momordica cochinchinensis	Arg198,Gln266,Arg258,Arg137	Arg198,Gln266,Arg258,Arg137	Gln205,Ala259,Ala210,Ala211,Val134	Gln205,Ala259,Ala210,Ala211,Val134	-8.8	-9.1

Table S4. Results of LDL lowering drug PCSK9 molecular docking based on positive control

Drug Category	Generic name	Wild type	Mutant type
		Docking energy, ΔG (kcal mol−1)	
Statins	Atorvastatin	-7.6	-7.9
Statins	Rosuvastatin	-7.1	-7.1
Statins	Simvastatin	-7.4	-7.3
Statins	Pravastatin	-7.1	-6.7
Cholesterol Absorption Inhibitors	Ezetimibe	-7.4	-7.3
Fibrates	Fenofibrate	-7.2	-7.3
Fibrates	Gemfibrozil	-6.2	-6.1
Nicotinic Acid Derivatives	Acipimox	-5.5	-5.4

Supplementary figures

Figure S1. Genetic variant distribution for serum LDL concentration risk in a genome-wide association study.

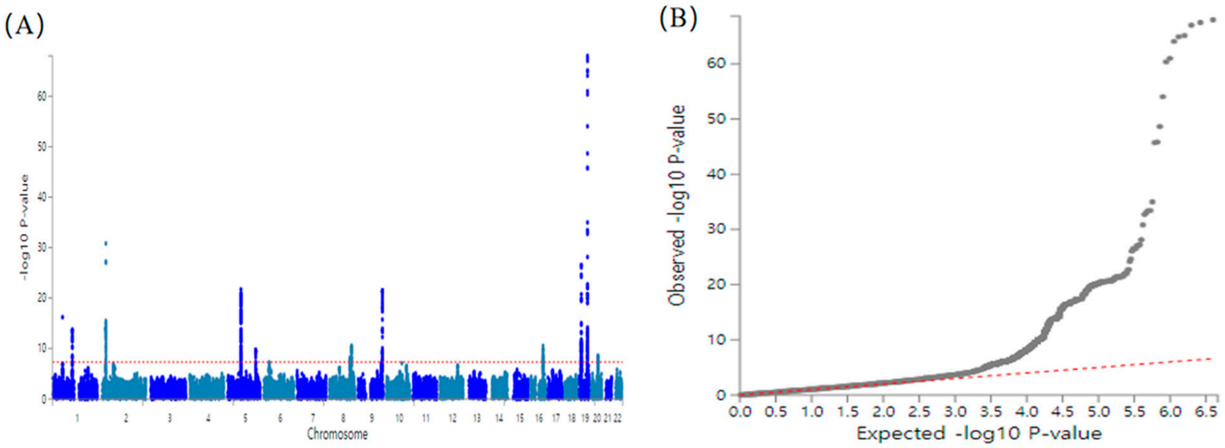

(A) Manhattan plot of the p-value of genetic variants. The red dotted line indicates the p-value of the cutoff of genetic variants for the serum LDL concentration (B) Q–Q plot of observed and expected p-values. The red dotted line indicated the calculated observed and expected p-value. It indicates the perfect matching between observed and expected p-values.

Figure S2. Adjusted odd ratios (OR) and 95% confidence intervals (CI) of hyper-LDL-cholesterolemia (excluding the participants with lipid-lowering medication) with genetic variants and their polygenic risk score (PRS).

A

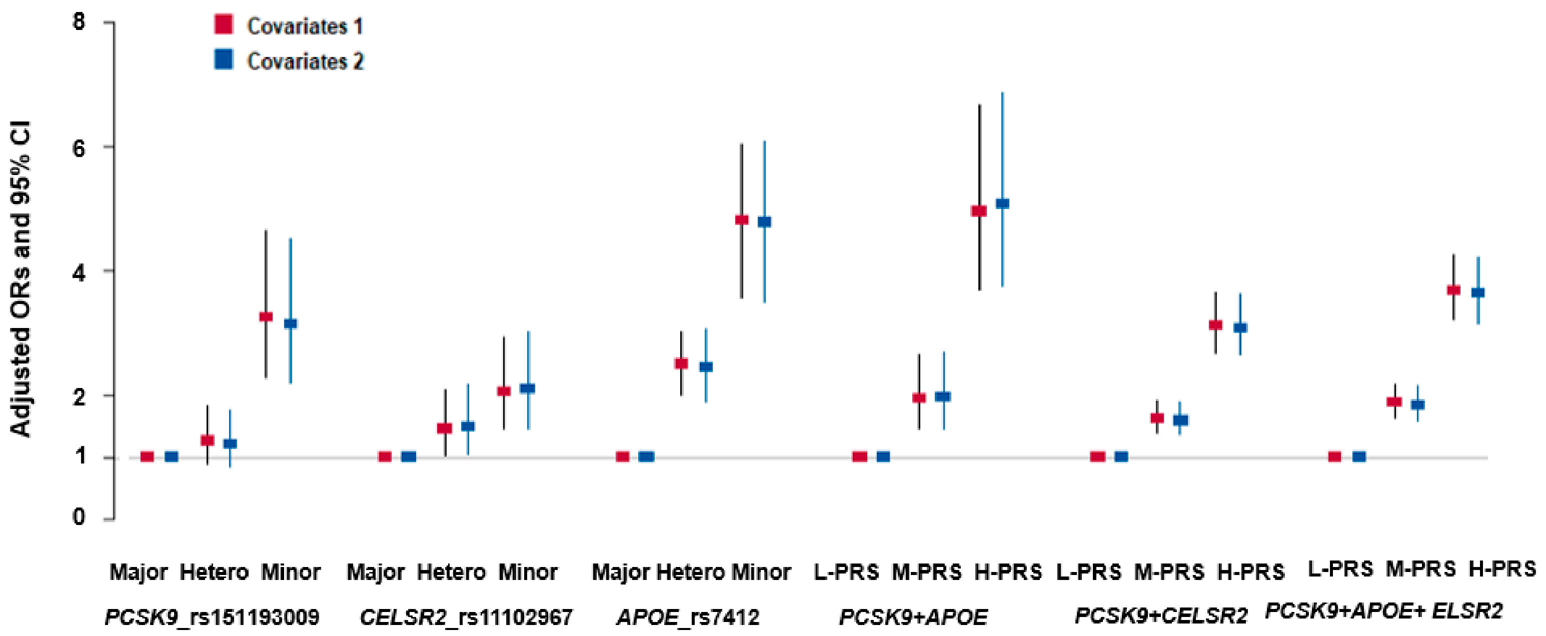

Figure S2B. Adjusted odd ratios (OR) and 95% confidence intervals (CI) of hyper-LDL-cholesterolemia (High-LDL defined as ≥130 mg/dL) with genetic variants and their polygenic risk score (PRS).

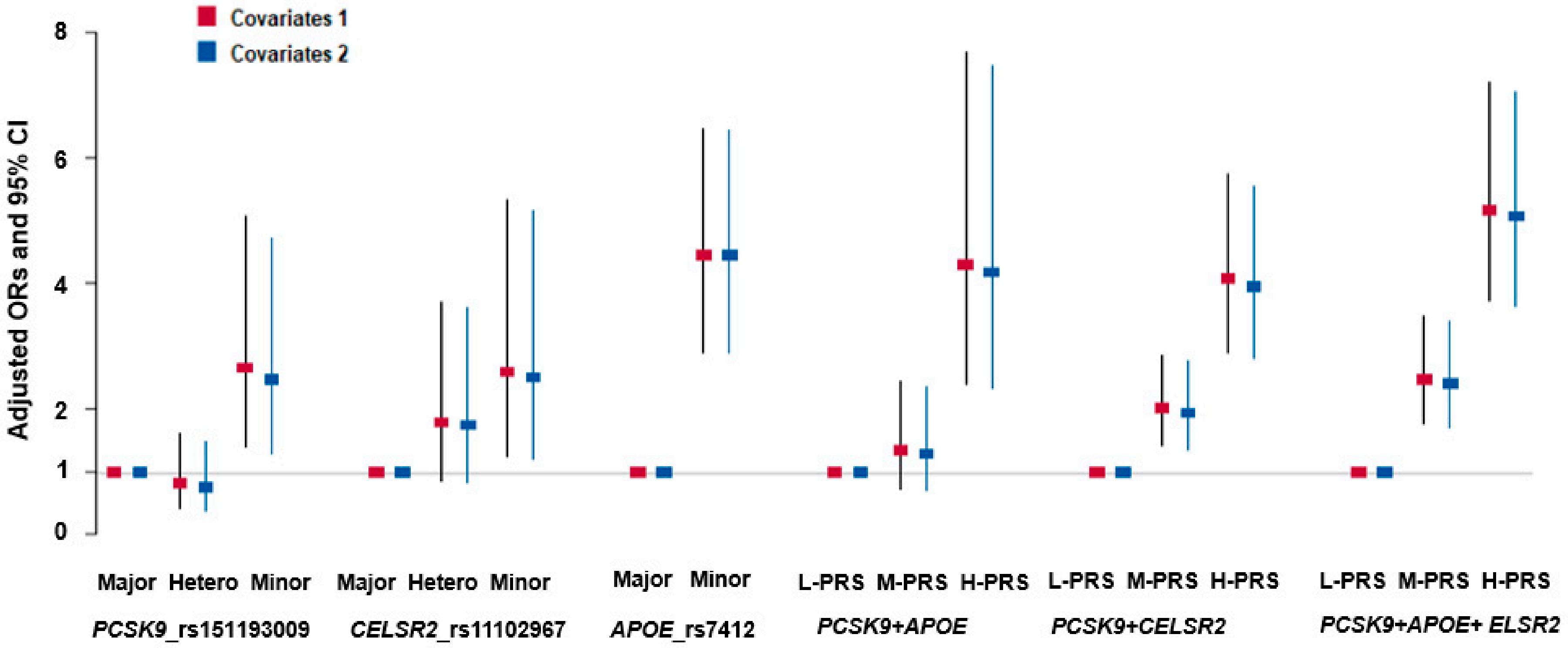

PRS was summing the number of risk alleles of *PCSK9_rs151193009, CELSR2_rs11102967,* and *APOE_rs7412.* Covariates 1: BMI, residence area, gender, age, and education; Covariates 2: covariates 1 plus energy intake, alcohol intake, physical activity, and smoking status.

Figure S3. Molecular docking of PCSK9_rs151193009 (Arg93) and MT (93Cys) with a bioactive compound for Pelargonidin 3-O-[2-O-(6-(E)-feruloyl-beta-D-glucopyranosyl)-6-O-(E)-p-coumaroyl-beta-D-glucopyranoside] 5-O-(beta-D-glucopyranoside)

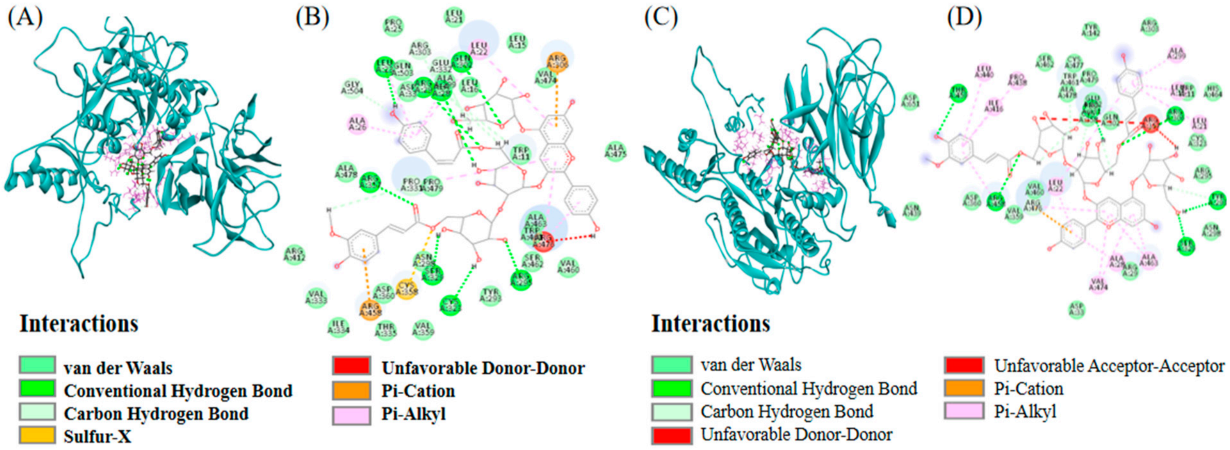

Figure S4. Molecular docking of PCSK9_rs151193009 (Arg93) and MT (93Cys) with a bioactive compound for 28-Glucosyloleanolic acid 3-[arabinosyl-(1->2)-6-methylglucuronide]

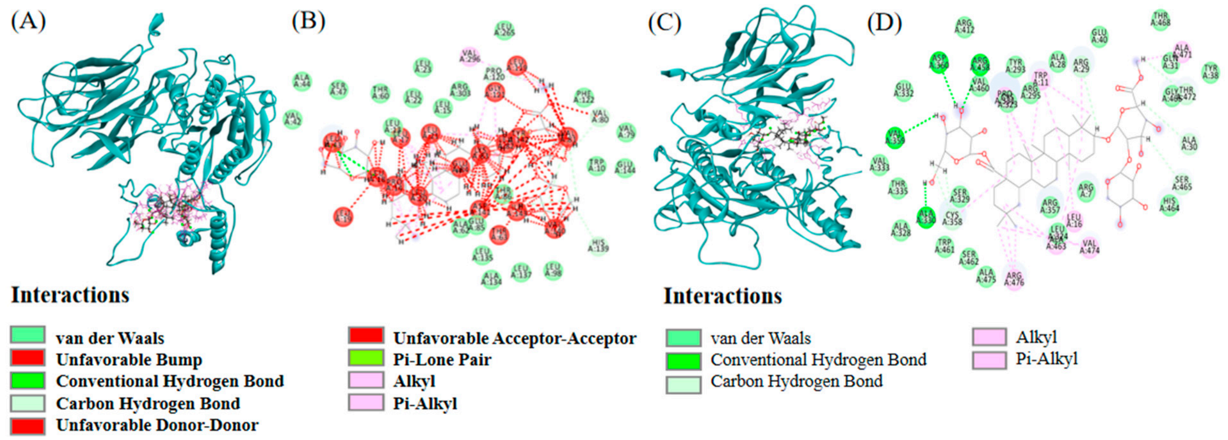

Figure S5. Molecular docking of PCSK9_rs151193009 (Arg93) and MT (93Cys) with a bioactive compound for Melongoside K

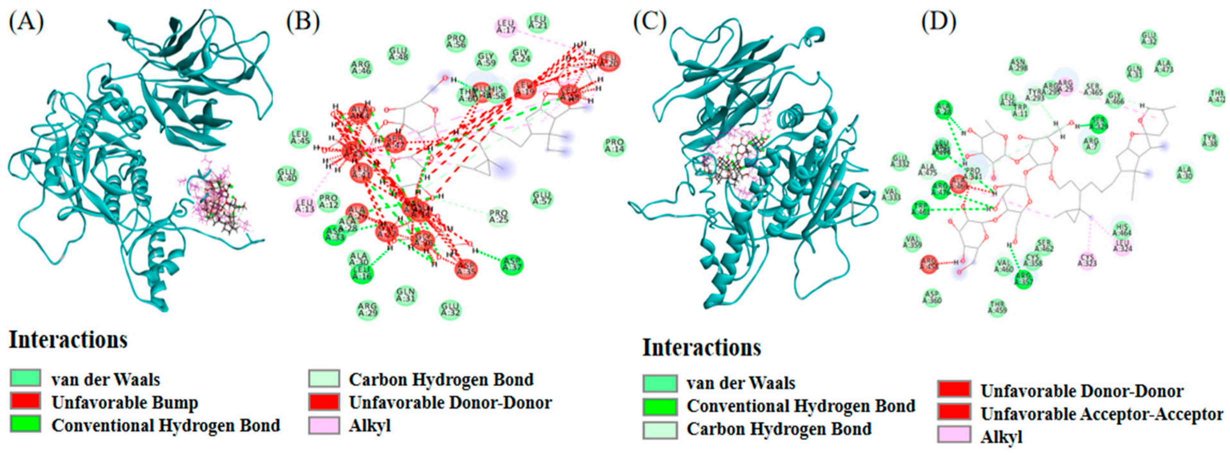

Figure S6. Molecular docking of PCSK9_rs151193009 (Arg93) and MT (93Cys) with a bioactive compound for Momordin IIa

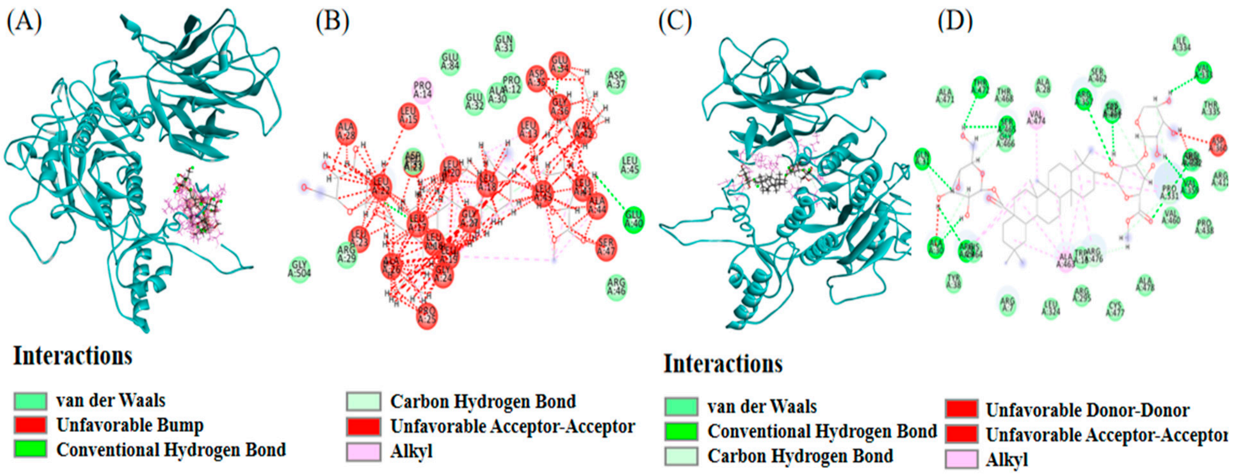

(A, C) Diagrammatic representation of the compound (ball and stick model) binding with WT and MT of Proprotein convertase subtilisin/kexin type 9 (PCSK9)

(B, D) 2D depiction of PCSK9 interacting with the compound and the nature of forces involve d in stabilizing–complex of bioactive compound WT and MT of PCSK9, respectively.

Figure S7. Molecular dynamics (MD) simulation of PCSK9_rs151193009 (Arg93) and MT (93Cys) and Pelargonidin 3-O-[2-O-(6-(E)-feruloyl-beta-D-glucopyranosyl)-6-O-(E)-p-coumaroyl-beta-D-glucopyranoside] 5-O-(beta-D-glucopyranoside) .

A.

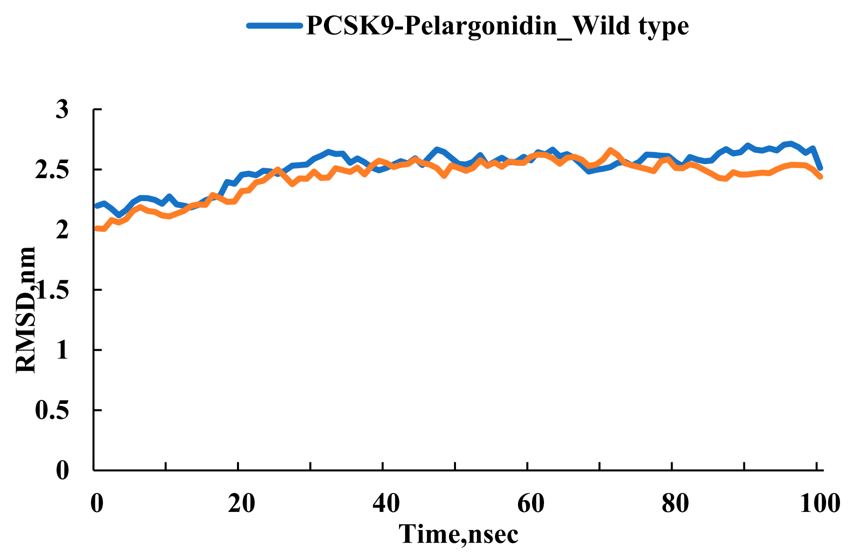

B.

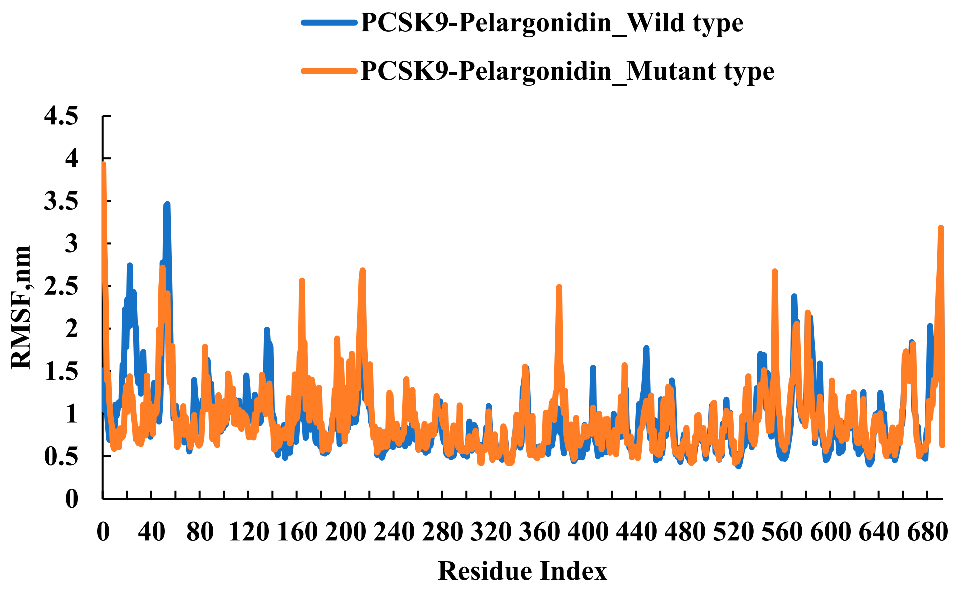

MD simulation of PCSK9_rs151193009 (Arg93) and MT (93Cys) and bioactive compound interaction. (A) Variation in root mean square deviation (RMSD) of PCSK9 alone and PCSK9–compound complex as a function of simulation; (B) Variation in root mean square fluctuation (RMSF) in PCSK9 in the absence and presence of compound.
